# Galectin-3: One Molecule for an Alphabet of Diseases, from A to Z

**DOI:** 10.3390/ijms19020379

**Published:** 2018-01-26

**Authors:** Salvatore Sciacchitano, Luca Lavra, Alessandra Morgante, Alessandra Ulivieri, Fiorenza Magi, Gian Paolo De Francesco, Carlo Bellotti, Leila B. Salehi, Alberto Ricci

**Affiliations:** 1 Department of Clinical and Molecular Medicine, Sapienza University, Policlinico Umberto I, Viale Regina Elena 324, 00161 Rome, Italy; alberto.ricci@uniroma1.it; 2 Laboratory of Biomedical Research, Niccolò Cusano University Foundation, Via Don Carlo Gnocchi 3, 00166 Rome, Italy; luca.lavra@fondazioneniccolocusano.it (L.L.); alessandra.morgante@fondazioneniccolocusano.it (A.M.); alessandra.ulivieri@fondazioneniccolocusano.it (A.U.); fiorenza.magi@fondazioneniccolocusano.it (F.M.); leila.b.salehi@fondazioneniccolocusano.it (L.B.S.); 3 Department of Oncological Science, Breast Unit, St Andrea University Hospital, Via di Grottarossa, 1035/39, 00189 Rome, Italy; Gianpaolo.defrancesco@gmail.com; 4 Operative Unit Surgery of Thyroid and Parathyroid, Sapienza University of Rome, S. Andrea Hospital, Via di Grottarossa, 1035/39, 00189 Rome, Italy; carlo_bellotti@hotmail.com; 5 Department of Biopathology and Diagnostic Imaging, Tor Vergata University, Via Montpellier 1, 00133 Rome, Italy

**Keywords:** Galectin-3, Gal-3

## Abstract

Galectin-3 (Gal-3) regulates basic cellular functions such as cell–cell and cell–matrix interactions, growth, proliferation, differentiation, and inflammation. It is not surprising, therefore, that this protein is involved in the pathogenesis of many relevant human diseases, including cancer, fibrosis, chronic inflammation and scarring affecting many different tissues. The papers published in the literature have progressively increased in number during the last decades, testifying the great interest given to this protein by numerous researchers involved in many different clinical contexts. Considering the crucial role exerted by Gal-3 in many different clinical conditions, Gal-3 is emerging as a new diagnostic, prognostic biomarker and as a new promising therapeutic target. The current review aims to extensively examine the studies published so far on the role of Gal-3 in all the clinical conditions and diseases, listed in alphabetical order, where it was analyzed.

## 1. Introduction

Galectins were initially isolated as β-galactoside-binding proteins. They represent a family of widely expressed lectins in metazoans. In mammalians a total of 15 galectins have been identified so far, containing either one or two ∼130 amino-acid-long conserved carbohydrate recognition domains (CRD)s [1,2,3]. Galectins are synthesized in the cytoplasm and interact with cell surface glycans following their secretion by a non-classical exocytic pathway (i.e., not via the ER/Golgi secretory route), that is likely to be an exosome-mediated secretory route [4,5]. Their ability to crosslink glycosylated ligands allows them to form a dynamic lattice. The galectin lattice regulates many different functions such as the diffusion, compartmentalization and endocytosis of plasma membrane glycoproteins and glycolipids, the selection, activation and arrest of T cells, receptor kinase signaling and the functionality of membrane receptors, (including the glucagon receptor, glucose and amino acid transporters, cadherins and integrins) [6].

**Figure 1:**
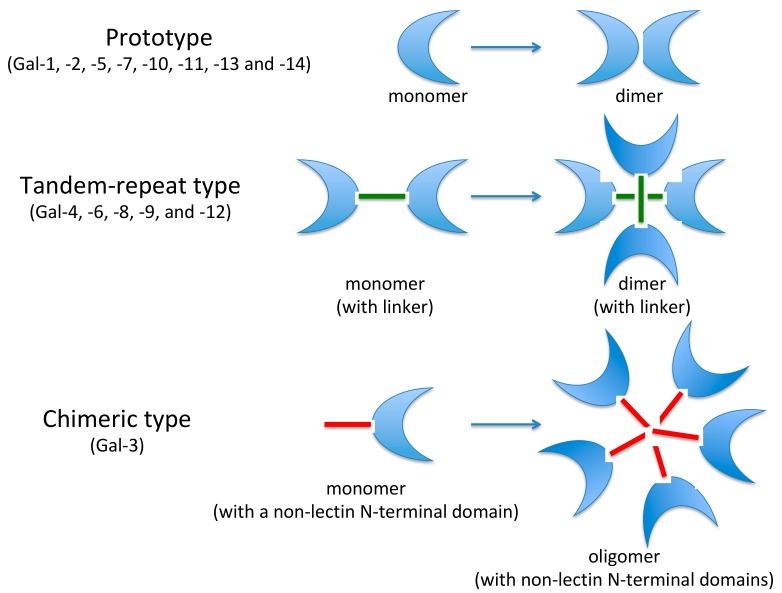
The structure of the galectin family members. The galectin family members are divided into three types: The prototype with one carbohydrate recognition domain (CRD), the tandem-repeat type with two CRDs connected by a non-conserved linker, and the chimeric type with one CRD and a non-lectin N-terminal domain (ND). Some galectins can self-associate into dimers or oligomers.

Although the galectins are cytosolic proteins, there is abundant evidence for their secretion from the cytosol via nonclassical pathways or translocation to the nucleus or the other cellular compartments [4,7]. The galectin family can be subdivided into three different groups:

1. Prototypic single-CRD galectins that can form non-covalent homodimers. The following galectins belong to this group: Gal-1, Gal-2, Gal-5, Gal-7, Gal-10, Gal-11, Gal-13, Gal-14 and Gal-15.
2. Tandem-repeats of two CRD motifs, similar but not identical, including Gal-4, Gal-6, Gal-8, Gal-9 and Gal-12.
3. The chimera-type. The only member of this group is represented by Gal-3, with a single CRD and an intrinsically disordered sequence at the N-terminal domain that promotes oligomerization. In addition, Gal-3 is the only one to be able to pentamerize. It exhibits specific pleiotropic biological function, playing a key role in many physiological and pathological processes (Figure 1).

### 1.1. Gal-3 Identification

Gal-3 is a member of a growing family of β-galactoside binding lectins [8], with an approximate molecular weight of 30,000. It was originally identified in murine peritoneal macrophages and named MAC-2 [9]. Later, it was also described as CBP-35, a 35-kDa carbohydrate-binding protein in mouse 3T3 fibroblasts [10]. Since then it was detected in numerous type of tumoral tissues [11], basophilic leukemia cells [12], human and murine tumor cells [13,14], human lung [15] rat intestine [16] and human HeLa cells [17].

Gal-3 is encoded by the *LGALS3* Gene, which has previously been known under different names: IgE-Binding Protein [18], MAC-2 [9], Lectin L-29 [19], L-34 [20] CBP-30 [21], Galectin 3 (Gal-3), Advanced Glycation End-Product Receptor 3, Lectin, Galactoside-Binding, Soluble, 3, Carbohydrate-Binding Protein 35 (CBP 35) [22], Galactose-Specific Lectin 3, Laminin-Binding Protein [23], 35 KDa Lectin, Galactoside-Binding Protein, GALBP [12].

### 1.2. Gal-3 Tissue Distribution

In adults, Gal-3 is ubiquitously expressed, while experiments performed in mice demonstrated during embryogenesis its expression is tissue- and time-dependent. Although its expression is mainly related to the epithelial cells and myeloid/amoeboid cells, Gal-3 expression was detected in many different types of cells, including: Small intestinal epithelial cells, colonic epithelia, corneal and conjuctival epithelia, olfactory epithelium, epithelial cells of kidney, lung, thymus, breast, and prostate. It was also detected in ductal cells of salivary glands, pancreas, kidney, eye, in intrahepatic bile ducts, in fibroblasts, chondrocytes and osteoblasts, osteoclasts, keratinocytes, Schwann cells and gastric mucosa, as well as in the endothelial cells from various tissues and organs [24]. In addition, there are numerous data on Gal-3 expression in the cells involved in immune response, such as neutrophils, eosinophils, basophils and mast cells, Langerhans cells, dendritic cells, as well as monocytes and macrophages from different tissues. In some other cell types, such as lymphocytes, Gal-3 is not normally expressed, yet it expression can be induced by various stimuli [4,7]. Moreover, Gal-3 displays pathological expression in many tumors, such as those affecting the pancreas, the liver, the colonic mucosa, the breast, the lung, the prostate, the head and neck, the nervous system and the thyroid [24,25,26].

### 1.3. Gal-3 Protein/Gene Structure and Carbohydrate Binding

Gal-3 is the most studied member of the galectin family. It is the sole member of chimera-type family of galectins [27]. Gal-3 (m.w. 31 kDa) is found in solution as a monomer with two functional domains [22,28,29,30]. Gal-3 is so far unique in the family in having an extra long and flexible N-terminal domain consisting of 100–150 amino acid residues, according to species of origin, made up of repetitive sequence of nine amino acid residues rich in proline, glycine, tyrosine and glutamine and lacking charged or large side-chain hydrophobic residues [4,7,29,30]. The N-terminal domain contains sites for phosphorylation (Ser 6, Ser 12) [31,32] and other determinants important for the secretion of the lectin by a novel, nonclassical mechanism [33]. The C-terminus is the CRD, consisting of about 135 amino acid residues; this is what defines the molecule as a galectin. The structure of the *LGALS3* gene is consistent with the multi-domain organization of the protein. The gene for Gal-3 is composed of six exons and five introns (human locus 14q21-22). Exon I encodes the major part of the 5′ untranslated sequence mRNA. Exon II contains the remaining part of the 5′ untranslated sequence, the protein translation initiation site and the first six amino acids including the initial methionine. The repetitive sequence in the N-terminal half of the gene product is encoded within exon III. Exons IV, V and VI code the C-terminal half of the protein [7,34]. Gal-3, like most members of the galectin family, acts as a receptor for ligands containing poly-*N*-acetyllactosamine sequences which consist of many disaccharide units: Gal β1,4 GlcNAc bond to each other by β1,3 linkage. However, Gal-3 appears to have an increased affinity for the more complex oligosaccharides [4,34].

### 1.4. Gal-3 Subcellular Localization

Although Gal-3 is predominantly located in the cytoplasm, it has also been detected in the nucleus, on the cell surface and in the extracellular environment, suggesting a multifunctionality of this molecule.

Extracellular Gal-3 modulates important interactions between epithelial cells and extracellular matrix, and plays a role in the embryonic development of collecting ducts [35]. In contrast, intracellular Gal-3 is important for cell survival due to its ability to block the intrinsic apoptotic pathway [36,37]. In this regard, it has been demonstrated that cytoplasmic Gal-3 in cancer cells is usually associated with an aggressive phenotype, in opposition to nuclear Gal-3 [38].

Nuclear localization of Gal-3 was first described in in vitro proliferating fibroblasts [39,40,41], but not in fibroblasts with replicative deficiencies [42]. Nuclear Gal-3 is localized mainly in interchromatin spaces, at the border of condensed chromatin, on the dense fibrillar component, and at the periphery of the fibrillar centers of nucleoli [43].

### 1.5. Gal-3 Secretion

Gal-3 is synthesized on free ribosomes in the cytoplasm and lacks any signal sequence for translocation into the endoplasmic reticulum (ER) [4]. Although it does not traverse the endoplasmic reticulum/Golgi network, there is abundant evidence for Gal-3 also having an extracellular location. This protein has been shown to be secreted from cells by a novel, incompletely understood mechanism called ectocytosis, which is independent of the classical secretory pathway through the ER and Golgi system [4,44,45]. A short segment of the Gal-3 N-terminal sequence comprising residues 89–96 (Tyr-Pro-Ser-Ala-Pro-Gly-Ala-Tyr) has been found to play a critical role in Gal-3 secretion. However, this sequence is not sufficient on its own to cause the direct secretion of the CAT fusion protein, indicating that it is operative in the context of a large stretch of the N-terminal sequence of Gal-3 [33]. Immunohistochemical studies have indicated that the first step in Gal-3 secretion is its accumulation at the cytoplasmic side of the plasma membrane [44,45,46].

### 1.6. Gal-3 Ligands

There are several known ligands for Gal-3 [47]. They can be subdivided, according to their localization, into extracellular, intracellular and nuclear ligands.

#### 1.6.1. Gal-3 Extracellular Ligands

Gal-3 binds to glycosylated extracellular matrix components, including laminin, fibronectin, tenascin, chondroitin sulfate and Mac-2 binding protein [21,48,49,50,51]. Some cell-surface adhesion molecules, such as integrins, are also ligands for Gal-3 and galectins, through binding to the extracellular domains of one or both subunits of an integrin, may positively or negatively modulate integrin activation, and affect binding with extracellular ligands [52].

A major ligand for Gal-3 on mouse macrophage is the α-subunit of the integrin αMβ2, otherwise known as CD11b/18 [53,54]. Moreover, Gal-3 also interacts with integrin α1β1 via its CRD domain in a lactose dependent manner [55]. Gal-3 also seems to be an endogenous cross-linker of the CD98 antigen, leading to the activation of integrin-mediated adhesion [53].

#### 1.6.2. Gal-3 Intracellular Ligands

Gal-3 is also known to have an intracellular location and to interact with several proteins inside the cell, such as: Cytokeratins [56], CBP70 [57], Chrp [58], Gemin4 [59], Alix/AIP-1 [60], and Bcl-2 [61]. It is worth noting that almost all of the mentioned intracellular ligands interact with Gal-3 via protein–protein rather than lectin-glycoconjugate interactions. The only exception is the cytokeratins. In fact, it has been shown that cytokeratins of MCF7, as well as other human cells, carry a novel posttranslational modification, a glycan with a terminal α linked GalNAc and that these residues are recognized in vitro by mammalian Gal-3 [56].

#### 1.6.3. Gal-3 Nuclear Ligands

Nuclear Gal-3 can interact with Gemin-4, a component of a macromolecular complex containing approximately 15 polypeptides, among them SMN (survival of motor neuron) protein, Gemin-2, Gemin-3, some of the Sm core proteins of snRNPs and other as yet unidentified proteins. These complexes are implicated in processes directly or indirectly related to pre-mRNA splicing. The identification of Gemin-4 as an interacting partner of Gal-3 provides strong evidence that this galectin can play a role in splicesome assembly in vivo [59,60]. The screening of a Jurkat cell cDNA library, by the yeast two-hybrid method using Gal-3 as bait allowed the identification of another Gal-3 binding protein. It is a human homologue of ALG-2 linked protein x (Alix) or ALG-2 interacting protein-1 (AIP-1) [60]. This protein interacts with ALG-2, a calcium binding protein necessary for cell death induced by different stimuli. AIP-1 cooperates with ALG-2 in executing the calcium dependent requirements along the cell death pathway [62,63]. Alix/AIP-1 contains a proline, glycine, alanine and tyrosine rich sequence in the C-terminal region, which is highly homologous to the tandem repeat sequence in the N-terminal part of Gal-3 [60].

## 2. Role of Gal-3 in Different Clinical Conditions and Diseases (Listed in Alphabetical Order)

**Figure 2:**
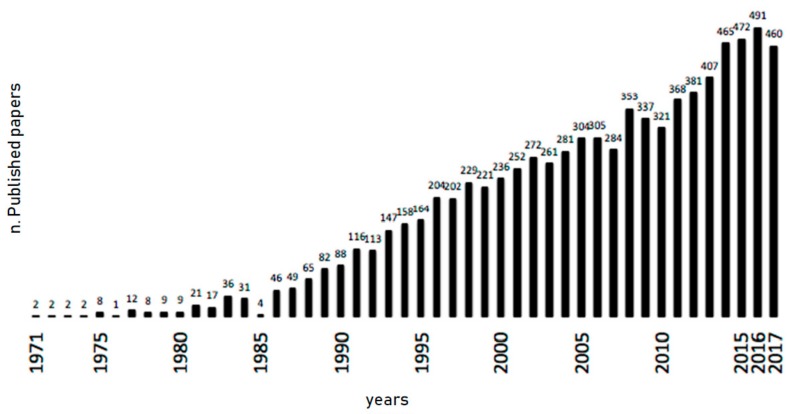
PubMed (U.S. National Library of Medicine, Medical Institutes of Health) records on academic publications on Gal-3, total 8289 items by December 2017. Search Strategy MESH terms: Galectin 3 or Gal-3 or Advanced Glycation End-Product Receptor 3 or Lectin, Galactoside-Binding, Soluble, 3 or Carbohydrate-Binding Protein 35 (CBP 35) or Galactose-Specific Lectin 3 or Laminin-Binding Protein or IgE-Binding Protein or Lectin L-29.

Gal-3 is a multifunctional protein engaged in different biological events, in different tissues, playing a relevant role in many different clinical conditions and diseases. Since its discovery as an IgE-binding protein in 1971 and subsequently its recognition as Mac-2, a 32-kDa cell surface antigen expressed on murine peritoneal macrophages in 1982, more than 8200 papers have been published in the literature so far (Figure 2).

The constant increase in the number of papers published in the most recent years and concerning the role of this protein in many clinical conditions and diseases testify the great clinical relevance of Gal-3 in human pathophysiology.

Numerous previous studies have indicated that Gal-3 may be used as a diagnostic or prognostic biomarker for many types of diseases and conditions. Recently, the potential clinical uses of Gal-3 as a diagnostic biomarker and as a therapeutic target were described in a comprehensive review [64]. 

**Figure 3:**
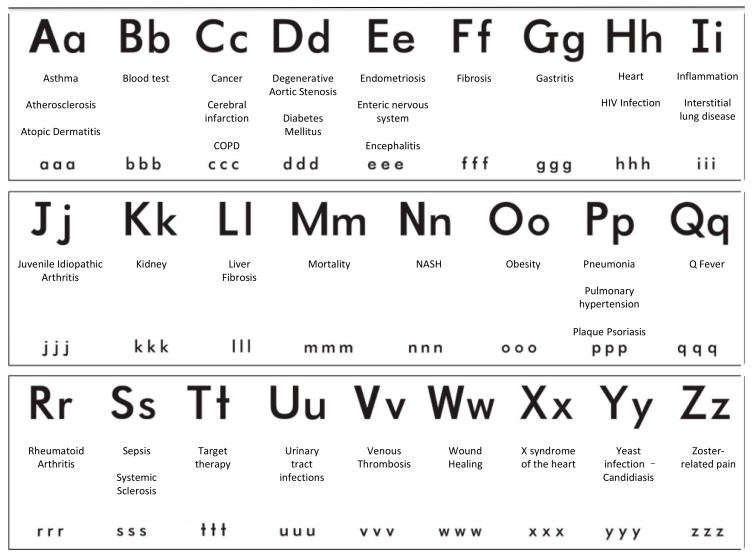
Conditions and diseases in which a role for Gal-3 has been postulated, listed in alphabetical order.

The current review represents an attempt to examine the many clinical implications of Gal-3 protein, based on a classification of clinical diseases, listed in alphabetical order (Figure 3).

## 3. A

### 3.1. Asthma

Asthma is a heterogeneous disease characterized by chronic airway inflammation, variable and reversible airway obstruction and airway hyperresponsiveness (AHR) [65]. Since their recognition, almost fifty years ago, the class of human antibody officially named IgE was unequivocally considered as the factor capable of transferring sensitivity to allergens and plays a central player in the allergic response [66]. It is now clear that IgE acts as part of a complex protein network. The interaction of genes belonging to such network with environmental factors is able to determine the risk of allergic sensitization [67]. In addition to the two major IgE receptors, namely the FcεRI (high affinity) and the FcεRII/CD23 (low affinity), Gal-3 has received increasing interest as a major player in this network and is generally thought to be potentially relevant in the pathogenesis of inflammation in asthma and its phenotypes. In this regard it is relevant to note that Gal-3 was initially identified as an IgE-binding protein [68]. Several experimental studies, using murine animal models, indicate that Gal-3 has a role in the pathophysiological mechanisms in immune response and, in particular, in asthma [69,70,71,72,73,74,75]. The specific role of Gal-3 in asthma is related to its function in the recruitment, activation and removal of neutrophils [73]. Experimental data from Gal-3 null mice showed reduced neutrophil recruitment during infection [74] and Gal-3 increases uptake of apoptotic neutrophils. Therefore, any Gal-3 dysfunction may influence the ability to remove apoptotic neutrophils from the site of inflammation [75]. This has several consequences: Reduction in the removal of apoptotic cells can result in the release of damaging enzymes and oxidants, which can promote persistence of inflammation. In humans, a significant reduced level of Gal-3 was detected in the sputum of neutrophilic asthma compared to eosinophilic and paucigranulocytic asthma [76]. However, the most interesting finding was the recent observation that Gal-3 may be considered as a new biomarker predictive of long-term responsiveness of pulmonary function in patients with severe asthma treated with the monoclonal antibody, anti-immunoglobulin E (IgE), named omalizumab [77]. According to this study, the analysis of bronchial biopsy specimens, obtained from patients with severe asthma, may help in predicting response to treatment. In particular, patients that were classified as Gal-3 positive before treatment initiation were responsive to the treatment with omalizumab while Gal-3 negative patients were non-responders to the same treatment with omalizumab. This study needs to be confirmed by others but it indicates that Gal-3 may be considered as new asthma-related biomarker, useful for patient stratification for therapy, in addition to the other biomarkers already known, such as the fractional exhaled nitric oxide (FeNO) levels, the blood/sputum eosinophil counts, the serum periostin levels, the smooth muscle proteins and the keratins.

Inflammation driven by type-2 helper T lymphocytes (Th2) is relevant in asthma and understanding the mechanisms that underline Th2 cytokine production is crucial for the establishment of novel therapeutic options. At present, there are limited data on the role of Gal-3 in allergic inflammation. As a multifunctional protein expressed by different types of inflammatory cells, Gal-3 has been considered as an IgE-binding protein and a regulator of infiltrating cell inflammation, activation and clearance [78]. Gal-3 activity may be operative in no-eosinophilic asthma [78]. Moreover, low levels of Gal-3 were seen in neutrophilic asthma compared with eosinophilic asthma [76]. Furthermore, galectins in relationship with their location (extracellular or intracellular, endogenous or exogenously delivered) may differentially regulate eosinophil behavior exerting a pro- or anti-inflammatory outcome. In severe asthma Gal-3 may be considered as a biomarker of lung remodeling and its levels are related to response to omalizumab treatment and improve lung function [77]. Moreover, Gal-3 deficient mice developed less airway hyperresponsiveness and eosinophils infiltration, a reduction of Th2 cytokines in the airways, during acute allergen-challenge if compared to WT mice [69,70,71], suggesting a role of Gal-3 in the chronic asthma. In Brown-Norway rats the intra-tracheal deposition of a vector with the gene encoding for Gal-3 causes the impairment of some Th2 effects, eosinophil influx and the down-regulation of IL-5 gene expression, IL5 that is one of the main regulatory cytokines that modulate eosinophilic behavior [79].

### 3.2. Atherosclerosis

Atherosclerosis is considered an inflammatory-proliferative response of the intima to injury [80,81]. The participation of monocytes in atherogenesis is well known [82,83] and their presence in the artery plaques and in the lipid core was recognized in 1993 [84]. More recently, macrophages were recognized as the principal inflammatory cell mediator in the atheromatous plaque that may participate in intima remodeling, immune response, and scavenger functions through the synthesis and secretion of a variety of enzymes and factors [80,85]. The role of Gal-3 in atherosclerosis was analyzed in specimens obtained from carotid endarterectomies, lower limb amputations, and thoracic aortas from autopsies of young adult trauma victims [86]. According to this study, a positive staining for Gal-3 can be detected close to a lipid core or near areas with fibrosis, neovascularization, calcification, hemorrhage, or thrombosis. In the carotid and lower limb arteries, foam cells were the cell type that most frequently stained positive for Gal-3. Mononuclear round cells and foam cells containing Gal-3 were also observed in the sub-endothelium, sometimes at sites of thrombosis. Endothelial cells and cells lining neo-vessels within plaques were not stained for Gal-3. Spindle cells from the tunica media of atherosclerotic arteries were not stained for Gal-3, but isolated Gal-3-positive cells were seen outside the lamina elastica externa. Gal-3 can be detected by immunoperoxidase staining in advanced atherosclerotic lesions of carotid and lower limb arteries and in early aortic atherosclerotic changes. In atherosclerotic lesions, Gal-3 is often localized in macrophages and foam cells and rarely in smooth muscle cells. Gal-3 is not detectable by immunohistochemistry in the tunica media of umbilical cord, carotid, limb, and aorta arteries. Finally, Gal-3 mRNA can be detected in advanced atherosclerotic lesions from carotid and lower limb arteries and a higher level of Gal-3 was demonstrated by Western blot analysis in these arteries in comparison with umbilical cord arteries.

The presence and the role of Gal-3 in most foam cells into the atherosclerotic lesions needs to be elucidated. It could be related to an association with lipid loading or alternatively the accumulation of Gal-3 together with advanced glycation endproducts (AGEs), may suggest that Gal-3 could act as a receptor for modified lipoproteins, thus participating in the formation of foam cells. More studies are needed to unravel any pathogenic or diagnostic role of Gal-3 in atherosclerosis.

### 3.3. Atopic Dermatitis

Atopic dermatitis (AD) is a chronic inflammatory skin condition that involves an inherited epidermal barrier defect, which may allow for greater penetration by allergens and irritants.

Atopic dermatitis, commonly called atopic eczema, usually arises in early childhood, often in infancy. While it usually resolves by early teenage years, approximately 5–10% of patients have the disease extend into adulthood. Classic symptoms are itching and burning of the skin, resulting in thickening of the skin in response to the scratching. In some adults, it can be severe with debilitating itching, inability to sleep, and social stigmatization due to skin damage and thickening, often on the face. IgE-associated atopic eczema/dermatitis syndrome (AEDS) contains abnormal B-cells that are surface IgE-positive (sIgE+) and produce IgE spontaneously in culture. Gal-3 is highly expressed in epithelial cells including keratinocytes, hair follicles, sebaceous, and sweat glands [87]. It has been reported that the IgE production was amplified by PMNs in patients AEDS, but not in healthy controls, and this effect was mediated by Gal-3 release by PMNs. In this regard, it has been demonstrated that IgE release was enhanced by the addition of Gal-3 to the cultures of AEDS B cells admixed with PMNs from healthy controls. These results suggest that Gal-3 can enhance PMN-induced IgE synthesis by B cells that are already committed to IgE production. These observations indicate that Gal-3 may be involved in the hyperactive immune response in AEDS [88]. Although initial evidence, based on the results of immunohistochemical analysis of skin biopsies from AD patients, argued against a critical role of Gal-3 in the binding of monoclonal or serum Ig-E [89], many experimental results obtained using mouse models of AD suggest that Gal-3 plays a critical role in the development of the allergic inflammatory response in AD [90]. The possible involvement of Gal-3 in the development of an allergic inflammatory response was, in fact, analyzed in an experimental mouse model in which AD was induced by repeated epicutaneous sensitization with ovalbumin (OVA) [90]. In this mouse model an upregulation of Gal-3 expression in the epidermis was observed after such sensitization. These results suggest that Gal-3 is a pro-inflammatory mediator of skin inflammation in atopic skin disease and that is required for the development of the Th2 inflammatory response to epicutaneously-introduced antigens through its effects on both DCs and T cells [90]. Based on these experimental results a company named “Galectin Therapeutics Inc.” (NASDAQ:GALT, Norcross, GA, USA) has developed a new Gal-3 inhibitor, capable of binding to Gal-3 and disrupting its function. A small, open label, investigator-initiated protocol was recently started and three adult patients with severe atopic dermatitis, classified as refractory, meaning that they have not responded to medical treatment beyond topical steroids, were successfully treated without any significant adverse reaction [91]. A phase 2 controlled trial, using various doses and regimens to further explore the drug’s potential, is now under way.

## 4. B

### Blood Test

Clinical parameters, such as advanced age, higher New York Heart Association (NYHA) functional class, reduced left ventricular ejection fraction (LVEF), lower body mass index, renal dysfunction, and anemia, have all been associated with poor outcomes in heart failure (HF), but are not significant predictors of mortality. Many efforts have been made recently to find biomarkers that might help in the risk stratification, and prognostication of acute and chronic heart failure beside the well-established markers, such as the brain natriuretic peptide (BNP) and its N-terminal part (NT-proBNP), the soluble suppression of tumorigenicity 2 (ST2), the growth differentiation factor-15, and the highly sensitive troponins.

In a pivotal paper published in 2004 by Sharma and colleagues [92] Gal-3 was identified as the most upregulated protein in an animal model of left ventricular hypertrophy (LVH) and HF suggesting that Gal-3 is a critical player in the pathogenesis and progression of HF. Since then an acceleration of research was observed, especially in the field of cardiology with many studies published concerning the role of Gal-3 in the evaluation and management of patients with heart failure. Gal-3 levels in plasma were, in fact, found to be positively associated with known cardiovascular risk factors, as age, sex, diabetes, hypertension, hypercholesterolemia, body mass index, renal function and smoking and was proposed as an additional prognostic factors, useful to identify high-risk patients in whom additional care management efforts and advanced therapies could be warranted.

Gal-3 was proposed in many studies as a new biomarker of heart fibrosis that could predict outcome of heart failure (HF) [93] and, in particular, occurrence of mortality [94,95]. In several cohorts of acute HF [96,97], as well as chronic HF [94], Gal-3 was shown to be a powerful predictor of mortality. Clinical effectiveness and prognostic value of this test have been demonstrated in several heart-failure trials, including the “Coordinating Study Evaluating Outcomes of Advising and Counseling in Heart Failure (COACH)” [97], the “Pro-BNP Investigation of Dyspnea in the Emergency Department (PRIDE)” [98], the University of Maryland Pro-BNP for Diagnosis and Prognosis in Patients Presenting with Dyspnea (UMD)” [99] and the “Heart Failure: A Controlled Trial Investigating Outcomes of Exercise Training (HF-ACTION)” [100]. Gal-3 was also associated with mortality when evaluated in the general population, as reported in the “Prevention of Renal and Vascular End-Stage Disease (PREVEND) study [101], in the “Framingham Offspring Study” [102] and in the “Rancho Bernardo Study” [103].

Finally, Gal-3 was associated with increased risk of developing AF in the Framingham study, but this association was not found to be significant after adjustment for traditional clinical AF risk factors [104].

Gal-3 is currently used in clinical practice and it is measured using many different blood assays by manual or automated assays. The enzyme linked immunosorbent assay manual assay (ELISA) is the most frequently used method in the published studies. Recently, FDA has approved for market new automated blood assays, with faster delivery of the results, the BGM Gal-3 assay, manufactured by BG Medicine, Inc. (Waltham, MA, USA) and the ARCHITECT Gal-3 assay, by Abbott Diagnostics (Abbott Park, IL, USA) and more are on their ways. According to this tests plasma levels lower than 17.8 ng/mL indicate low risk, levels greater than 17.8 ng/mL but lower than 25.9 ng/mL are indicative of moderate risk and levels above 25.9 ng/mL are associated with high risk of adverse outcomes and need more frequent follow-up and intensified as well as advanced strategies of cure. Plasma Gal-3 levels greater than 25.9 ng/mL, independent of symptoms, clinical findings, and other laboratory measures, predict a patient who is likely to have rapid progression of heart failure, eventually resulting in hospitalization and death [105].

## 5. C

### 5.1. Cancer

A large body of reported data about galectin expression in cancer is available in the literature and the chapter on Gal-3 action on cancer development and progression represents one of the most relevant ones. Gal-3 has been shown to influence many significant biological processes linked to cancer development and progression, including cell adhesion, proliferation, differentiation, mRNA splicing, cell-cycle progression, immune system evasion, inflammation, angiogenesis, apoptosis and metastasis [25,38,106,107,108,109,110]. In addition, Gal-3 exerts a role as a pro-tumor factor by acting within the tumor microenvironment, the so-called “tumor niche”, to suppress immune surveillance. Its ability to promote tumor cell proliferation and survival of the cancer cell both directly and indirectly, by acting on cell adhesion and cell contact with mesenchymal stromal cells, has lead to consider Gal-3 as a guardian of the tumor microenvironment [111].

Gal-3 influences oncogenesis, cancer progression and metastasis through a variety of pathways both inside and outside the cell [112,113,114,115,116,117,118]. The studies on the role of Gal-3 in different types of cancers indicate that such effects may be specific for the different types of cancer. In fact, Gal-3 may have both positive and negative effects on cancer cell survival depending of the type of tumor considered and its subcellular localization likely influences the role of Gal-3 in different cells [24,25,26].

#### 5.1.1. Gal-3 Subcellular Localization in Tumor Tissues

In tumor tissues Gal-3 can be detected in the nucleus [119,120], in the cytoplasm [121], and eventually can be secreted into the extracellular spaces [4] or in the circulation [122]. The localization of Gal-3 is essential to its function. Gal-3’s function, in fact, will vary depending on whether it is in the nucleus, cytoplasm, or extracellular spaces [123]. In addition, as observed for Gal-1, the effects of Gal-3 seem to be cell type specific [24,119,124,125,126]. All these factors make extremely difficult to simplify the role of Gal-3 in cancer and may explain the variable and sometimes opposite effects reported in different cells and in different cellular compartments.

#### 5.1.2. Gal-3 and Apoptosis in Tumor Tissues

The most intensively studied mechanism through which Gal-3 influence cancer cell is represented by its anti-apoptotic function [24,61,110,127]. The elucidation of the mechanisms through which Gal-3 exerts its effect on apoptosis has led to identify a new apoptotic pathway in which different types of DNA damage triggers the HIPK2 activation and induces p53 mediated repression of Gal-3 expression [128]. Dysregulation of this pathway due to the occurrence of p53 mutations or due to HIPK2 loss may alter such pathway thus leading to Gal-3 overexpression and [129]. Gal-3 influences also many other signal transduction cascades and pro-survival processes that include major oncogenes such as *RAS*, *BCL2*, and *MYC* [130,131,132,133,134,135,136,137].

The ability of Gal-3 to form lattices with glycoproteins and glycolipids has been implicated in regulating cell adhesion, metastasis, endocytosis, and other biological processes [138,139,140,141,142,143]. Aberrant glycosylation could influence transport and trafficking of many different proteins involved in cancer development and progression [143]. Interaction of Gal-3 with glycosphingolipid (GSL) resulted in invaginations in the membrane that occurred during the endocytotic process and has been referred to as “membrane bending” [140,141,142,143]. “Membrane bending” requires both Gal-3 and GSL. As of now, integrin β1 endocytosis is mediated by GSL and Gal-3 and it is likely that other glycoproteins will also be transported by this mechanism [143].

#### 5.1.3. Gal-3 Immune Surveillance and Angiogenesis in Tumor Tissues

Gal-3 is able to modulate a variety of immune cell processes by co-opting selected inhibitory receptors, disrupting co-stimulatory pathways and/or controlling activation, differentiation, and survival of immune cells. On the other hand, Gal-3 can regulate vascular signaling programs through binding to integrin avb3 or by sustaining the pro-angiogenic capacity of tumor-associated macrophages. Therefore, Gal-3 serves as a bifunctional messenger that has emerged as a novel regulatory checkpoint able to couple immunosuppression and angiogenesis, two events that occur simultaneously during tumor growth and are often mediated by the same cytokines and the same cell types [144].

#### 5.1.4. Gal-3 in Various Types of Human Cancers

The large body of studies, regarding the expression of Gal-3 in various types of cancers that have been published in the literature were obtained using different types of cells/tissues as well as various animal models [38,106,145]. The specific techniques used, the inclusion of appropriate controls and the heterogeneity of the tissues examined may explain the discrepancies observed in the different studies. It is relevant to note that the percentage of normal epithelial, stromal, vascular and inflammatory cells may constitute a strong bias in the evaluation of the expression of Gal-3 in a given cancerous tissue specimens. This is especially true for Gal-3, a protein that is highly expressed in inflammatory cells. In general, histological techniques such as immunohistochemistry or in situ hybridization that allow a cell type-specific analysis of Gal-3 expression are most reliable, compared to Western blot and RT-PCR techniques that may be give results that are potentially contaminated or diluted by the presence of noncancerous cells. Gal-3 was analyzed as oncologic marker both by immunohistochemistry and by measuring its blood levels.

An increased expression of Gal-3 was observed at immunohistochemistry or at RT-PCR, in many different types of cancers [25,146,147] and most of the authors agree that detection of Gal-3 could help to better define diagnosis and/or prognosis of cancer. Increased expression of Gal-3 was reported breast carcinoma, in colon carcinomas, in gastric cancers, in hepatocellular carcinoma, in well differentiated thyroid carcinomas in anaplastic large-cell lymphoma, in head and neck squamous cell carcinomas, in tongue carcinomas and in non-small cell lung cancer. Other studies demonstrated an opposite picture, with decreased Gal-3 expression in breast, ovarian cancer prostate tumors, advanced uterine adenocarcinoma, basal cell carcinoma of the skin, epithelial skin cancer and malignant salivary gland neoplasms, compared to the corresponding normal tissue. Several studies have investigated the expression of Gal-3 in in human colon carcinomas, but conflicting results have been reported [148,149,150,151]. It seems that Gal-3 expression is down-regulated in the initial stages of colon neoplastic progression [152,153], whereas a dissociated cytoplasmic expression increases in later phases of tumor progression [154].

Among the cancers that express high level of Gal-3 there are those affecting the thyroid gland [155,156,157,158,159,160,161], the central nervous system in adults [162] and in children [163,164], the head and neck squamous cell [165], the pancreas [166], the stomach [112], the bladder [167], the kidney [168,169], the liver [170], in the parathyroid [171,172] and in the salivary glands [173]. In addition, elevated Gal-3 expression levels were also observed in lymphoma [174] and in melanoma [175]. Finally, Gal-3 was found to play a relevant prognostic role in neuroblastoma [164].

The analysis of the expression levels of Gal-3 was extensively studied in the thyroid tissue. In this tissue, Gal-3 expression analysis was suggested as a presurgical marker of cancer [156] and the detection of Gal-3 expression, specifically located in the cytoplasm of the thyroid cells was used a diagnostic hallmark of thyroid malignancy. On such basis a ThyroTests was setup [176,177,178], clinically validated [160,179] and used in many countries [180]. Its good accuracy and its low cost has led to propose its inclusion in the diagnostic algorithm of thyroid cancer as a screening test especially useful for suspicious thyroid nodules with a cytologically indeterminate pattern [181,182].

In addition to the analysis of the expression levels in neoplastic tissues, Gal-3 was also investigated as a potential new oncologic scintigraphic as well as blood marker in many different types of tumors, including the thyroid [183,184,185], the prostate [186] and the ovarian cancer [187].

### 5.2. Cerebral Infarction

It is often difficult to determine the prognosis and clarify the pathophysiology of patients referred to emergency department for suspected ischemic stroke. Computerized cranial tomography (CCT) and diffusion-weighted magnetic resonance imaging (MRI) are crucial in the diagnosis of cerebral infarction. However, a lot of efforts have been made to search for new biomarkers and, in particular, for serum markers specifically associated with brain damage, that may allow discrimination of ischemic stroke and intracerebral hemorrhage. The levels of Gal-3 have been reported to increase in ischemic brain damage [188,189]. In a recent study, Gal-3 serum levels were found to be increased in clinically-suspected ischemic stroke patients with normal CCT, seen in the emergency department. On such basis, it has been suggested that its measurement may contribute to the diagnosis of cerebral infarction [190]. Such promising studies need to be confirmed in large numbers of patients.

### 5.3. Chronic Obstructive Pulmonary Disease

At present there are limited data available on the possible role of Gal-3 in chronic obstructive pulmonary disease (COPD). COPD is a chronic, progressive, irreversible disease associated with high morbidity and mortality. It is characterized by bronchial epithelial changes and neutrophil infiltration. Gal-3 has been demonstrated to be higher in patients with COPD with elevated systolic pulmonary artery pressure compared to healthy controls [191]. In this subset of patients, Gal-3 levels seem to be a predictive marker of right ventricular dysfunction [191]. In small airways of severe COPD patients, epithelial Gal-3 immunostaining was increased when compared with healthy nonsmokers or smokers [192]. In addition, in patients with severe COPD, Gal-3 expression and neutrophil accumulation in the small airway epithelium correlate with epithelial proliferation and airway obstruction [192]. Finally, in the airway of current or ex-smoker COPD patients, Gal-3 and its receptor CD98 were decreased. Furthermore, Gal-3 levels measured in broncho alveolar lavage (BAL) were reduced in healthy smokers suggesting a potential role of smoke in regulating Gal-3 expression although in COPD ex-smokers the defect in Gal-3 expression persists despite cessation of smoking [193]. Administration of exogenous Gal-3 to alveolar macrophages from BAL, cultivated in vitro and obtained from patients affected by COPD, caused an increase in efferocytosis, the process by which dying/dead cells (e.g., apoptotic or necrotic) are removed by phagocytic cells, thus indicating a possible role of Gal-3 as a novel macrophage-targeted therapies for COPD/Emphysema [194]. Very recently, the level of serum Gal-3 was significantly increased in acute exacerbation of COPD compared with the level in COPD convalescence phase, suggesting that Gal-3 might be a valuable diagnostic biomarker for this condition [195]. In such patients, the serum levels of Gal-3 positively correlated with systemic inflammation and smoking [195].

## 6. D

### 6.1. Degenerative Aortic Stenosis

The first clinical report concerning the relationship between myocardial Gal-3 expression and severe aortic stenosis (AS) was published in 2004 [92]. In this study, Gal-3 expression was analyzed in cardiac biopsies from patients undergoing aortic valve replacement for aortic stenosis. Gal-3 myocardial expression was increased in aortic stenosis patients with cardiac hypertrophy and relatively depressed ejection fraction compared to aortic stenosis subjects with LV hypertrophy and normal or elevated ejection fraction.

Recently, another study was conducted to test the potential prognostic value of Gal-3 in patients with degenerative AS [196]. In patients treated with balloon aortic valvuloplasty (BAV) Gal-3 was associated with worse survival and this was unrelated to estimated glomerular filtration rate (eGFR). Measurement of circulating levels of Gal-3 may therefore be useful in risk stratification in elderly patients affected by degenerative AS with coexistent diseases to choose the optimal therapy.

### 6.2. Diabetes Mellitus

Whereas most of the experiments revealed a protective role of Gal-3 in acute inflammation, its function in chronic inflammatory diseases, like diabetes mellitus (DM) and in particular in type 2 diabetes mellitus (T2DM) is less clear [197,198]. The role of Gal-3 in the development of Diabetes of its complication is still an area of debate. Several studies indicate that Gal-3 is involved in the regulation of glucose homeostasis by acting at the level of adipose tissue and pancreatic islets, thus participating in the pathogenesis of obesity and T2DM, but its effects may be influenced by differences in genetic background, diet, age, sex, as well as experimental end points. In fact, in the literature controversial results have been reported regarding to the roles of Gal-3 in the pathogenesis of T2DM. In some studies Gal-3 was considered as a protective factor, while in others it was found to be associated with an increase in the severity of T2DM.

#### 6.2.1. Gal-3 Increases Severity of DM

In humans, systemic Gal-3 is elevated in obesity and negatively correlates with glycated hemoglobin in T2DM patients [199]. In a population-based cross-sectional survey in Zhejiang, China involving large number of patients, high Gal-3 serum levels were associated with increased odds of developing heart failure, nephropathy, and peripheral arterial disease in patients with T2DM [200]. In other clinical studies, circulating Gal-3 was higher in T2DM patients [201,202,203] and thought to be a risk factor for vascular complications, such as heart failure, nephropathy, peripheral artery disease, and other vascular complications. Patients with Gal-3 level > 25 ng/mL exhibited a 11.4-fold higher risk of microvascular complications (retinopathy and/or nephropathy) and a 8.5-fold increased risk of macrovascular complications (myocardial infraction, angina pectoris, cerebrovascular event, and peripheral artery disease) compared to patients with Gal-3 level < 10 ng/mL [200]. The order of circulating level of glectin-3 was as follows: Diabetes > prediabetes > normal control. Therefore, it has since been considered as a marker for prediabetes [201]. In diabetic patients, Gal-3 concentrations were significantly elevated in subjects with coronary artery disease and associated with the formation of diseased vessels and plaques [203]. In agreement with human studies, also in mice models in which hyperglycemia was induced after high-fat diet feeding with a corresponding threefold increase in homeostasis model assessment for insulin resistance (HOMA-IR) index and a 1.5-fold decrease in Akt activation, indicate that Gal-3 expression was increased in endothelial cells as well as in blood, suggesting that Gal-3 plays a role in the vascular response in DM [204]. Another report found increased Gal-3 expression in mice fed with a high-fat diet and recombinant human Gal-3 promoted preadipocyte proliferation [205]. Moreover, galecitin-3 knockout mice exhibited less weight gain when fed with a high-fat diet. The expression levels of PPAR-γ, CCAAT-enhancer-binding protein alpha (C/EBPα), CCAAT-enhancer-binding protein beta (C/EBPβ), and fatty acid binding protein 4 (Fabp4) were also reduced in the adipose tissue of Gal-3 knockout mice fed with a high-fat diet [206]. Gal-3 upregulation was also found in hepatocytes treated with 100 mM d-glucose and in the sera of patients with T2DM [207]. Consistently, Gal-3 ablation protected mice from the development of streptozotocin-induced type 1 DM (T1DM) [208].

#### 6.2.2. Gal-3 Decreases Severity of DM

Although most of the studies agree in considering Gal-3 as a marker of inflammation and fibrosis, many studies suggest that the increased expression of Gal-3 may be part of an adaptive response to tissue injury, favoring resolution of inflammation and opposing to chronification of the inflammatory process [209]. Both clinical studies in humans and experimental studies in mice, indicate that Gal-3 may indeed play a protective role in some metabolic diseases, such as T2DM. In a study of 20 patients, Ohkura et al. measured the glucose disposal rate, fasting insulin, HOMA-IR, insulin sensitivity index, Gal-3, and adiponectin. Notably, Gal-3 levels negatively correlated with fasting insulin and HOMA-IR, but positively correlated with glucose disposal rate, insulin sensitivity index, and serum adiponectin level [210]. Moreover, body weight, amount of total visceral adipose tissue, fasting blood glucose, and insulin levels increased in high-fat diet-fed Gal-3 knockout mice. These mice also displayed enhanced Th1 T-cells and natural killer T (NKT) cells, as well as pro-inflammatory CD11c^+^CD11b^+^ macrophages, whereas the number of anti- inflammatory CD4^+^CD25^+^FoxP3^+^ regulatory T-cells and M2 macrophages were reduced. Infiltrating pancreatic and peritoneal macrophages also exhibited significant increases in NLR family, pyrin domain containing 3 (NLRP3) inflammasome, nuclear factor-κB activity, and IL-1β production [211]. Gal-3 deficiency in mice led to the accumulation of fat as well as increased inflammation in adipose tissue, which is important in promoting insulin resistance. There were higher numbers of infiltrated macrophages and inflammatory cytokines, IL-6 and TNF-α, in the adipose tissue. High-fat diet also attenuated the expression of adiponectin and PPAR-γ in the adipose tissue [212]. Furthermore, galecitin-3 deficiency was also found to suppress endothelial glucose transporter, type 4 (GLUT4) expression and promote insulin resistance in high-fat diet-fed mice, as suggested by comparing Gal-3 knockout mice to wild-type controls [213]. Experimental studies, performed in mice, demonstrated a protective role of Gal-3 toward obesity and T2DM, via the modulation of the responsiveness of innate and adaptive immunity to overnutrition [212,214]. In accordance with such hypothesis, Gal-3 KO in mice induces a pro-inflammatory phenotype characterized by an increased systemic, pancreatic and VAT inflammatory response to metabolic stimuli and an exacerbated vascular and renal tissue damage induced by diabetes and related disorders [215,216]. Moreover, Gal-3 deficient mice exhibit greater hyperglycemia and impaired glucose tolerance compared to control wild type mice, indicating that Gal-3 deficiency contributes to the pathogenesis of diabetes [213].

The conflicting data reported so far regarding the role Gal-3 in obesity and T2DM suggest that we need of further attention and more clinical studies in order to clarify its role as a potential player and therapeutic target in these conditions.

## 7. E

### 7.1. Endometriosis

Endometriosis is an inflammatory disease of reproductive-aged women, and it is strongly related to consequent infertility [217]. Endometriosis is caused by a dysregulation of inflammatory and vascular signaling [218]. Gal-3 plays a relevant role in both these processes and it is overexpressed in endometriotic tissue [219]. In addition, higher levels of Gal-3 can be detected in the peritoneal fluids from women with endometriosis [220]. Another aspect in which Gal-3 may be involved in the pathogenesis of endometriosis and its complication in the development of pain associated with inflammation. Gal-3 was found to be involved in myelin phagocytosis, in Wallerian degeneration of neurons, and is able to trigger neuronal apoptosis after nerve injury [221]. It has been hypothesized that Gal-3, overexpressed in endometriotic foci, could be responsible for the induction of nerve degeneration and pain [222]. In this regard it is interesting to note that neurotrophin, a nerve growth factor strongly expressed in endometriosis, upregulates galcetin-3 expression [219]. All of these studies suggest that Gal-3, and other Galectins as well, play important roles in immune responses in inflammatory disease and in the pathogenesis of endometriosis, but there is still need of high-quality clinical studies to confirm these observations.

### 7.2. Enteric Nervous System

The enteric nervous system (ENS) governs the function of the gastrointestinal (GI) tract. It is embedded in the lining of the gastrointestinal system, beginning in the esophagus and extending down to the anus [223]. It is derived from neural crest cells and is often named as the second brain [224,225]. Dysfunction of ENS, due to ischemic cerebral stroke, may be responsible for the occurrence of neurological manifestation that complicate stroke, including dysphagia, dysmotility and colorectal dysfunction.

It is well known that Gal-3 in the GI tract stabilizes cell-cell junctions and enables polarization of intestinal epithelial cells [226,227]. Furthermore, it has been associated with GI cancers [228], as well as with Crohn’s disease and ulcerative colitis [229].

Induction of stroke in experimental murine models by permanent middle cerebral artery occlusion (pMCAO) triggers central and peripheral Gal-3 release by microglia. Increased levels of Gal-3 are, in turn, responsible for enteric neuronal loss [230]. Gal-3, therefore, appears to be a crucial mediator of the GI damage observed after brain injuries, directly at the level of enteric neuronal cells and it may represent a potential new target for the treatment of post-stroke gastrointestinal complications. In this regard, it has been recently reported that elevated serum levels of Gal-3 correlates with poor outcome after acute heart failure and high Gal-3 values are observed in heart failure patients with a history of stroke [231].

### 7.3. Encephalitis

Gal-3 is expressed in a variety of glial cells of the central nervous system (CNS) tissues [232] and contributes to the migration of neuroblasts during brain development [233] and to the differentiation of oligodendrocytes [232]. Since Gal-3 is involved in inflammatory response and in the interaction with extracellular matrix, a potential role has been postulated in various types of CNS inflammation and in particular in viral encephalitis. Its expression was analyzed in some neuroinflammation animal models. It has been reported that Gal-3 is upregulated after long-term virus inoculation in a *Junin virus*-induced encephalitis model using newborn mice [234]. Gal-3 is rapidly upregulated in activated microglia, in the *encephalomyocarditis virus*-induced adult mouse encephalitis model of viral infection [235]. Gal-3 was found to be expressed in macrophages in remote spinal cord dorsal horn lesions caused by peripheral inoculation of *herpes virus* [236].

## 8. F

### Fibrosis

Tissue fibrosis is characterized by the occurrence of tissue overgrowth, hardening, and/or scarring [237]. Tissue fibrosis may progressively evolve toward a severely debilitating disease, characterized by superabundant accumulation of extracellular matrix (ECM) including collagen. Tissue fibrosis represents the end result of chronic inflammatory reactions induced by a variety of stimuli including persistent infections, autoimmune reactions, allergic responses, chemical insults, radiation, and tissue injury and ultimately may lead to excessive tissue scarring, organ injury, function decline, and even failure [238,239,240]. Fibrosis is generally considered a major cause of morbidity and mortality worldwide because therapeutic options for the treatment of tissue fibrosis are rather limited. The only effective treatment in end-stage fibrotic disease is often represented by the organ transplantation. It is well-known that the mechanisms driving fibrogenesis are distinct from those regulating acute inflammatory reactions but few clear pathogenic and therapeutic targets have been identified so far. Recently, Gal-3 has emerged as a key regulator of chronic inflammation and tissue fibrogenesis and as an attractive new therapeutic target in the search for effective anti-fibrotic therapies [241].

Many studies indicate that Gal-3 is increased in patients with fibrosis affecting different tissues. In particular, Gal-3 is associated with fibrosis affecting the liver [170,242], the kidney [243,244], the lung [245,246], the heart [247,248] as well as the nervous system [249]. In addition, Gal-3 was found to be associated with systemic sclerosis (SSc), and in particular with skin fibrosis and proliferative vasculopathy observed in such condition [250]. Recently, another study indicated that the serum Gal-3 levels were higher in the SSc patients group compared with the controls group and they were higher in the active SSc group than in the inactive SSc group [251]. These data suggest that Gal-3 may be considered as a prominent biomarker of disease activity in SSc. In these studies examining the pathogenic role of Gal-3 in fibrosis, Gal-3 is identified as the link between macrophages, fibroblasts and the pro-fibrotic phenotype. It is known, in fact, that Gal-3 mediates IL-4 induced alternative macrophage activation and that IL-4/IL-13 activated macrophages upregulate profibrotic genes, stimulating matrix production and enhancing fibrosis [252,253,254]. These observations have led to the concept of a Gal-3/macrophage/fibroblast “axis”.

On the basis of such observations regarding the profibrotic role of Gal-3, the measurement of its serum levels was proposed as a new prognostic factor [101]. In the Prevention of REnal and Vascular ENd-stage Disease (PREVEND), serum Gal-3 levels were measured in 7968 subjects that were followed-up for of approximately 10 years. Gal-3 serum levels were correlated with established risk factors for CV disease and mortality in subjects from the general population and gradually increased with age. After correction for such classical CV risk factors (smoking, blood pressure, cholesterol and diabetes) the fibrosis marker Gal-3 independently predicted all-cause mortality. The same observation was recently reported for patients with systemic sclerosis where Gal-3 was found to be the best predictor of the all-cause mortality in a group of 152 patients with systemic sclerosis that were followed-up for up to 10 years [255]. All these results indicate that Gal-3 may be considered as a useful new simple biomarker in the prediction of future CV events and mortality and its inhibition may represent a promising therapeutic strategy against tissue fibrosis.

## 9. G

### Gastritis

Numerous infectious agents, such as *Trypanosma cruzi* [256], *human immunodeficiency virus-1* [257] and the *human T-lymphotropic virus-1* [258], have been reported to be able to induce Gal-3 upregulation. The first report regarding the association between Gal-3 and gastritis due to *Helicobacter pylori* infection was published in 2006 [259]. This study demonstrated that the expression of Gal-3 was upregulated by gastric epithelial cells following adhesion of *Helicobacter pylori*, suggesting that in addition to colonization this protein also plays a role in the host response to infection. Murine experimental models have recently confirmed the key role played by Gal-3 in the innate immunity against infection and in the colonization of gastric mucosa by *Helicobacter pylori* [260]. Administration of extracellular recombinant Gal-3 (rGal-3) was shown to inhibit the adhesion of H. pylori to the gastric epithelial cells resulting in a decrease in apoptosis. Gal-3 acts as a chemoattractant in the recruitment of THP-1 monocytes, working as a negative regulator of *Helicobacter pylori* infection and its effects on gastric mucosa [261]. Since there is a strong association between *Helicobacter pylori* infection and the development of gastric adenocarcinoma and mucosa-associated gastric lymphoma [262,263], it has been suggested that the expression and upregulation of Gal-3 may act a critical endogenous event in *Helicobacter pylori* infection that interferes with various intracellular events, causing prolonged cell survival, which is characteristic in carcinogenesis [264].

## 10. H

### 10.1. Heart

Although Gal-3 is expressed at a low constitutive expression level in the heart, under pathophysiologic conditions, the level of expression of Gal-3 may change substantially and, therefore, may play a prominent function.

The first observation regarding the role of Gal-3 in heart failure was published in 2004 [92]. In this study, performed on a rat model of severe hypertension and overt heart failure, the Gal-3 was the strongest regulated gene, being overexpressed in decompensated hearts. A more than five-fold upregulation was observed in rat with decompensated hearts compared to those with compensated hearts. These results were confirmed in human heart tissues, in the same study. Gal-3 expression analysis, performed in ventricular biopsies from patients with aortic stenosis with preserved versus depressed ejection fraction demonstrated an upregulation in the myocardial biopsies from patients with depressed ejection fraction.

The demonstration that Gal-3 is responsible for the induction of left ventricular remodeling and development of heart failure came from experimental studies, using rats in which Gal-3 was infused in the pericardial sac [265]. The structural and functional modifications of the rat myocardial tissues were prevented by the co-administration of *N*-acetyl-seryl-aspartyl-lysyl-proline (Ac-SDKP), a naturally occurring tetrapeptide, acting as a Gal-3 inhibitor. The mechanism of action of Gal-3 at the myocardium level is not entirely clarified yet and it may be a general event in left ventricular dysfunction caused by different etiologies. Once Gal-3 is overexpressed it is responsible for activation of fibroblasts and macrophages and consequent induction of fibrosis, scar production and, ultimately, of cardiac remodeling. Gal-3 appears to be involved in the control of interstitial fibrosis, especially in the overloaded hearts [266]. The demonstration of the role of myocardial Gal-3 expression in heart failure has prompted some authors to evaluate the possibility that Gal-3 serum levels may be useful in the diagnosis of acute heart failure in patients presenting with dyspnea [96]. According to what suggested by Kramer [267], all the clinical trials evaluating Gal-3 in cardiovascular disease patients can be categorized into four groups of studies, according to their aim:

• assessing the utility of the serum/plasma concentration of the lectin for diagnosis,
• helping in stratifying patients for therapy,
• monitoring therapy response, and
• predicting short- and long-term morbidity and mortality.

The clinical utility of Gal-3 in the diagnosis of heart failure was not clearly demonstrated and, in particular, Gal-3 did not show correlation with the NYHA functional classification of patients and, in addition, it showed lower specificity and sensitivity in identifying heart failure, as compare to the NT-proBNP [96,268]. Gal-3 proved to be more useful in as a marker for patient stratification for therapy. Low levels of serum Gal-3 (<19 ng/mL) were associated with better survival rates and lower cardiovascular event rates in patients treated with rosuvastatin therapy [269]. Other evidence regarding the clinical utility of Gal-3 in stratification for therapy came from the Multicenter Automatic Defibrillator Implantation Trial with Cardiac Resynchronization Therapy (MADIT-CRT) study [270]. In this study, patients with high baseline Gal-3 levels (in the top quartile of the distribution) benefited more (65% reduction in risk of the primary end point) from cardiac resynchronization therapy compared to those with lower baseline Gal-3 values (25% decrease in risk). The clinical utility of Gal-3 measurement in the serum as a marker for monitoring therapy was evaluated in patients that underwent to left-ventricular assist device (LVAD) implantation [271,272]. In such patients, however, unloading of the heart did not influence plasma Gal-3 levels within the first 30 days after device implantation. Gal-3, therefore, did not provide sufficient discrimination for prediction of outcome.

Much more promising are the studies regarding the clinical utility of Gal-3 measurement in the serum in predicting short- and long-term morbidity and mortality [94,101,105,273,274]. Many different clinical trials have been conducted so far to test the prognostic value of Gal-3 in patients with heart failure as well as in the general population. Gal-3 proved to be associated with mortality in 232 patients with chronic heart failure, followed for 6.5 years. Gal-3, therefore, represents a significant predictor of mortality risk in patients with heart failure, after adjustment for age and sex, and severity of HF and renal dysfunction, as assessed by NT-proBNP and estimated glomerular filtration rate, respectively [94]. On the basis of such evidence the Gal-3 blood test was proposed in the evaluation of patients with heart failure [105] and in the management of acute heart failure patients presenting to the emergency department [275]. Much interest came form the Prevention of REnal and Vascular END stage disease (PREVEND) clinical trial, the largest study with the longest observation period, aimed to investigate Gal-3 levels to date [101]. In such study a total of 7968 Caucasian individuals from the general population were examined and followed up for 10 years. It is interesting to note that Gal-3 serum levels increase with age and that females showed higher values than males. This study demonstrated that there is a clear association of Gal-3 serum levels and increased all-cause mortality, suggesting that measurement of Gal-3 in the serum may be useful not only to predict outcome in patients with heart failure but also in the general population.

At the present time, current guidelines indicate that the measurement of biomarkers of fibrosis, such as soluble ST2 and Gal-3, may be considered for additive risk stratification (class of recommendation IIa, level of evidence A) [276,277,278].

### 10.2. HIV

*Human immunodeficiency virus* (HIV) triggers decreases in both the number and function of CD4^+^ T lymphocytes. Its replication cycle consists of three stages: Attachment and entry, gene and protein expression and assembly and budding. Gal-3 was found to play a role in HIV-1 viral budding [279]. After infection HIV-1 can survive for a long period without gene expression in host cells or can cause continuous viral expression without cytopathic effects on the cells. HIV-1 principally targets CD4^+^ T cells and cells of the monocyte/macrophage lineage [280].

A specific role of Gal-3 expression in HIV infection was hypothesized many years ago. Gal-3 was found to be associated with early stages of HIV infection, in particular during transport and/or splicing of HIV mRNA. Gal-3 expression greatly increases after infection of Molt-3 cells with HIV type 1, concomitantly with the onset of expression of Tat protein [281]. The increase in Gal-3 expression was mediated at the transcriptional level. Gal-3 promoter, in fact, was significantly upregulated by expression vectors encoding the 40-kDa Tax protein, a potent transactivator in HTLV-1 [258]. Subsequent studies demonstrated that Tat protein expression is able to induce an upregulation of Gal-3 in several human cell lines, by means of Tat transactivation of Sp-1-rich regulatory sequences upstream of the Gal-3 gene [257]. The role of Gal-3 in HIV infection however, is not entirely elucidated yet. The effect on HIV infection, however, seems to be different, and perhaps opposite, from those exerted by Gal-1 [282]. Recently, it has been suggested that Gal-3 may be involved in the host response to HIV infection. According to this study, Gal-3 is induced in HIV-1-infected macrophages and may promote death of HIV-1/SIV-infected macrophages, thus contributing to eradicate HIV infection [283].

## 11. I

### 11.1. Inflammation

Besides its known expression in epithelial and endothelial cells, Gal-3 is also expressed in many different cells involved in mediating inflammatory response, such as macrophages [284], monocytes [285], dendritic cells [286], eosinophils [287], mast cells [288], natural killer cells [289], as well as T lymphocytes [290] and activated B lymphocytic cells [291]. In addition, Gal-3 is overexpressed in phagocytic macrophages and is, therefore, considered as a macrophage activation marker [292]. The role of Gal-3 in inflammation has been extensively reviewed in many studies [197,291,293,294,295].

Gal-3 is involved in many processes during the acute inflammatory response including neutrophil activation and adhesion [296], chemoattraction of monocytes⁄macrophages [285], opsonization of apoptotic neutrophils [75], and activation of mast cells [297]. Gal-3 is abundantly expressed and secreted by macrophages [298]. Furthermore, expression of Gal-3 is increased when monocytes differentiate into macrophages [298] and decreased when immature dendritic cells differentiate into mature dendritic cells [299]. Gal-3 also augments monocyte–monocyte interactions that lead to polykaryon (multinucleated giant cell) formation, a phenotype associated with alternative macrophage activation [300].

Since the role of neutrophils is considered crucial with respect to the immune response to microbial agents, several studies have investigated also the relationship between Gal-3 and neutrophils activation and migration. By using a murine pneumonia model system, Gal-3 was found to be accumulated into the alveolar space, following induction of pneumonia with *Streptococcus pneumoniae* and that this event correlated with the onset of neutrophil extravasation [301]. Interestingly, the accumulation of Gal-3 in lung was not observed during neutrophil emigration into alveoli induced by *Escherichia coli* infection. Thus, this effect appears to be specific for the alveolar infection caused by *Streptococcus pneumoniae*.

In addition, Gal-3 was found to display antimicrobial activity against the fungus *Candida albicans* [302].

Gal-3 effects on monocyte–monocyte interactions are also involved in chronic inflammatory and fibrotic diseases [303]. However, its role is complex and both pro-fibrotic and protective roles for Gal-3 have been reported in models of chronic inflammation and fibrogenesis. To further complicate the story, the effects of Gal-3 appear to be dependent on both the organ and type of injury model analyzed.

Chronic inflammation with resultant fibrosis, loss of tissue architecture, scarring and subsequent organ failure characterizes the pathogenesis of many chronic human diseases and is a major cause of morbidity and mortality. Tissue fibrosis is mediated by the fibroblasts and myofibroblasts, which represent key cells in the initiation and perpetuation of tissue fibrogenesis [304] and by the macrophages themselves, which are involved, in the same time, in both the evolution and resolution of tissue fibrosis [305]. Gal-3 and Gal-1 are potent mitogens for fibroblasts in vitro. Gal-3 is able to stimulate DNA synthesis and cell proliferation in quiescent cultures of normal human lung fibroblast [306] as well as in fibroblast from different tissues [307].

### 11.2. Interstitial Lung Disease

Interstitial lung diseases are a wide group of lung diseases in which the pathologic process involves the interstitium. Among these diseases, idiopathic pulmonary fibrosis (IPF) represents a separate nosographic entity, characterized by rapid progression toward lung failure and dead. IPF is characterized by restriction in lung capacity, due to scarring of lung tissue. IPF is an orphan condition that affects between 200,000 and 300,000 subjects in the Western world. Current therapies have little effect on the natural course of the disease and only partial treatment options are available. There are few studies in experimental mouse models and in humans on the role of Gal-3 addressing this issue. Gal-3 was increased in a mouse model of bleomycin induced lung fibrosis [308]. In humans, Gal-3 was increased in the serum of patients affected by IPF, and higher Gal-3 concentrations were associated with decreased lung volumes and altered gas exchange [309]. Furthermore, the increase of Gal-3 has been documented in the broncho-alveolar lavage fluid (BALF) from patients affected with IPF and this increase appear to be specific for IPF and interstitial pneumonia associated with collagen vascular disease (CVD-IP) because patients with other interstitial lung disease such as the cryptogenic organizing pneumonia/bronchiolitis obliterance organizing pneumonia (COP/BOOP), the acute hypersensitive pneumonia and the *Pneumocystis jiroveci* infection did not show any significant increase of Gal-3 in the BAL fluid [246]. The increase in Gal-3, measured in the BAL fluid was associated with an increase of Gal-3 protein expression inside the alveolar macrophages, detected by immunofluorescence. Gal-3 is produced and secreted by alveolar macrophages and its induction may be determined by pro-inflammatory cytokines. It is well known that TNF alpha, a pro-fibrogenetic cytokine, is able to induce in vitro Gal-3 production by macrophage/monocyte cell line model [310,311]. A positive feedback between TNF alpha and Gal-3 was hypothesized based on the ability of Gal-3 to induce TNF alpha production sustaining inflammation [246]. It was also reported that Gal-3 might participate in vascular remodeling, known to be present in fibrotic lung [312]. In this regard, Gal-3 was able to induce capillary tube formation of endothelial cells in vitro and to stimulate angiogenesis in vivo [313]. Gal-3 action on neovascularization observed in IPF may be due to both a direct effect on endothelial cells and an indirect effect through IL 8 production [246]. Based on the assumption that Gal-3 is responsible for a promoting effect on IPF and considering the lack of effective treatment for this pulmonary condition, it has been postulated that Gal-3 may represent a possible new target for treatment. According to this hypothesis, the inhibition of Gal-3 expression and activity would result in an amelioration of the clinical conditions of patients affected. Taking advantage of a new compound named TD139, a highly potent, specific inhibitor of the galactoside binding pocket of Gal-3 a novel target therapy for IPF was proposed and a clinical trial process was started in 2014. TD139 was formulated for inhalation, which enables direct targeting the fibrotic tissue in the lungs, while minimizing systemic exposure. A randomized, double-blind, multi-centre, placebo-controlled, multiple dose expansion cohort study, was designed to assess the safety, tolerability, PK and PD of TD139 (ClinicalTrials.gov Identifier: NCT02257177). Galecto Biotech Company recently announced the successful completion of phase Ib/IIa clinical trial of inhaled TD139, administered to human volunteers and IPF patients. The possibility to treat IPF by targeting Gal-3 molecule offer the chance to better treatment of such devastating disease and open a new era on the clinical use of Gal-3 antagonists.

## 12. J

### Juvenile Idiopathic Arthritis

Juvenile idiopathic arthritis (JIA) is characterized by hyperplasia of synovial cells and accumulation of mononuclear inflammatory infiltrates. Such accumulation is generally thought to be due to the alteration of the local balance between cell proliferation and apoptosis. In agreement with this hypothesis, patients with polyarticular juvenile arthritis showed low apoptotic indexes, accompanied by low Bcl-2 expression, low proliferation rates and upregulation of Gal-3 expression in their synovial tissue [314]. Gal-3 upregulation in the synovial tissues was suggested to be the cause of the defective mononuclear apoptosis observed in synovial inflammatory infiltrates from these patients. Other evidence derives from the studies that have measured the serum levels in patients affected by JIA. Gal-3 serum levels were increased in a group of 50 active JIA children, compared to control subjects [315]. In addition, a positive correlation was demonstrated between serum Gal-3 levels and disease activity, i.e., the higher the level during exacerbations the higher it remained after remission. Finally, in such juvenile patients, the synovial fluid Gal-3 levels were found to be increased (approximately three times higher) compared to the serum level in paired samples. All these observations indicate that Gal-3 plays a crucial role in the pathophysiology of Juvenile idiopathic arthritis. Further studies including larger number of patients are needed, but it can be postulated that Gal-3 would be used in the future as a new biomarker for JIA disease, indicating activity, severity and progression of disease.

## 13. K

### Kidney

Gal-3 is expressed during embryogenesis and regulates branching morphogenesis and metanephrogenesis [316]. Gal-3 plays a role also in the adult human kidney [317,318]. In normal adult kidneys, its expression is restricted to the collecting tubules and primary cilium. However, it has been reported in rats that its expression can be temporarily re-induced during regeneration after tissue injury [319]. Experimental studies, performed in mice and rats, demonstrated that Gal-3 is markedly upregulated in acute tubular injury and the subsequent regeneration [319] as well as in progressive renal fibrosis [243]. Gal-3, indeed, appears to be a potent activator of fibroblasts in the kidneys [320]. However, its absence is protective against renal myofibroblast accumulation and activation [243]. Many studies, performed on mouse models of diabetic nephropathy and acute renal failure, concordantly indicate that Gal-3 is upregulated in such conditions [215,243,319]. Interesting results were obtained in studies performed also in humans with renal failure and especially with those affected by diabetes. Gal3, in fact, was found to be upregulated in the glomeruli of diabetic patients and the number of Gal-3 positive cells in the glomeruli correlated with the observed urinary protein excretion [321], suggesting a potential prognostic role of Gal-3 in predicting a poor outcome of such patients. In this regard, one of the most interesting observation regarding the correlation between Gal-3 and kidney function comes from the LUdwigshafen RIsk and Cardiovascular health (LURIC) study, based on data from 2578 patients [322] and from 4D studies based on data obtained from 1168 patients [323]. The analysis of Gal-3 plasma levels, measured in patients from these two large prospective studies, demonstrated a significant association of Gal-3 levels with cardiovascular end points, infections, and all-cause death in patients with impaired renal function [324]. The correlation was observed during a period of follow-up extended to 10 years (LURIC study) or four years (4D study) and the increase of Gal-3 levels was observed in parallel with the decrease in kidney function, with a marked elevation in dialysis patients affected by T2DM. Based on such evidence, Gal-3 was proposed a novel treatment opportunity for renal disease. Kolatsi-Joannou et al. investigated changes in the expression of Gal-3 in mice with acute kidney injury induced by folic acid, using modified citrus pectin (MCP), a pectin derivative that can bind to the Gal-3 carbohydrate recognition domain [325]. At two weeks of the recovery phase, MCP-treated mice demonstrated reduced Gal-3, with decreased renal fibrosis, macrophages, pro-inflammatory cytokine expression, and apoptosis. Their findings reveal that pharmacologic inhibition of Gal-3 with MCP was protective in experimental nephropathy by modulating early proliferation and later, Gal-3 expression, apoptosis, and fibrosis. Thus, MCP may be considered and proposed as a novel target to reduce long-term renal injuries, attenuate renal fibrosis and preserve kidney function, possibly via the effect of Gal-3 on carbohydrate binding-related functions [325]. A recent phase II study comparing treatment with another Gal-3 inhibitor, namely the GCS-100, and a placebo in patients with CKD stage 3b showed that GCS-100 significantly improved the eGFR [326]. Taken together, Gal-3 may be used to assess the prognosis, guide therapy, and potentially suggest specific anti-Gal-3 therapy. However, prospective studies are needed to validate these hypotheses.

## 14. L

### Liver Fibrosis

Liver fibrosis represents the final pathway of chronic damage to the liver, in conjunction with the accumulation of extracellular matrix proteins [327]. Chronic inflammation with the formation of scar tissue, loss of tissue architecture, and organ failure is a characteristic feature of this disease. An increased in Gal-3 expression has been previously reported in fibrosis in different types of tissues [170,308,328]. The main causes of liver fibrosis include chronic HCV infection, alcohol abuse, and nonalcoholic steatohepatitis (NASH). Hepatic stellate cells (HSCs) are considered the main collagen-producing cells in the liver. Gal-3 was found to play a critical role in the regulation of HSC activation, both in vitro and in vivo [329]. In particular, the HSC activation to a myofibroblast phenotype, a critical event in extracellular matrix deposition and in the development of cirrhosis, is dependent on the upregulation of Gal-3 expression within liver cells, in the areas of fibrotic tissue injury [327]. Based on such experimental and clinical evidence, a Phase 2b NASH-CX trial is ongoing (ClinicalTrials.gov Identifier: NCT02462967) to treat NASH cirrhosis patients without esophageal varices [330]. It is a randomized, double-blind, placebo-controlled clinical trial in patients with NASH cirrhosis without varices and is aimed to target Gal-3 expression by means of an antagonist developed by Galectin Therapeutics Inc. (Norcross, GA, USA) and named GR-MD-02. Preliminary results, released by the company on 2 December 2017, indicate a positive trend in treated patients versus placebo control patients, with an improvement in portal hypertension or liver biopsy. The results of this trial could lead to a better treatment of such condition and to effective prevention of its progression toward cirrhosis.

## 15. M

### Mortality

Many different studies have reported that Gal-3 concentrations are associated with incident HF and mortality in the general population and measurement of serum Gal-3 levels may be useful in predicting mortality for all-cause and cardiovascular disease (CVD) [101,102,103,331]. Gal-3, in fact, was a significant predictor of CVD mortality and of all-cause mortality in a large population study, based on 1393 participants without CVD with a mean age of 70 years, who were followed up for a mean of 11 years for the occurrence of coronary heart disease, CVD mortality, and all-cause mortality [103]. The same results were reported by the Framingham Heart Study, based on 3353 participants in the Framingham Offspring Cohort (mean age 59 years, 53% women), in which Gal-3 was associated with risk of all-cause mortality [102] and by the Prevention of Renal and Vascular ENd-stage Disease (PREVEND), based on 7968 subjects where Gal-3 levels independently predicted all-cause mortality [101].

## 16. N

### Non-Alcoholic Steatohepatitis (NASH)

Non-alcoholic steatohepatitis (NASH) is a condition in which fat builds up in the liver along with inflammation, composed predominantly of lymphocytes and Kupffer cells, and liver damage, in the absence of a history of alcoholism. It is more frequent in patients with obesity, metabolic syndrome and T2DM and is the most common chronic liver disease, with a rapidly increasing prevalence worldwide. The hallmarks of the disease are the presence, at histology, of steatosis, lobular inflammation, hepatocellular ballooning and fibrosis, which typically begin in zone 3 and show a characteristic perisinusoidal/pericellular (“chicken wire”) pattern [332]. A link between Gal-3 and hepatic fibrosis was established when mice that lack the Gal-3 gene were found to be resistant to liver fibrosis induced by toxin administration [242]. Such observations are in agreement with studies in other organs and systems regarding the role of Gal-3 in activating a variety of profibrotic factors, promoting fibroblast proliferation and transformation, and mediating collagen production [333]. The effects of Gal-3 knockout on NASH are rather controversial. It has been demonstrated that such mice are resistant to fat accumulation, inflammation and fibrosis when fed a high-fat diet [334]. However, other groups have demonstrated that Gal-3 null mice show an increased propensity to develop NASH [335]. An attempt to explain such discrepancy was recently given [336]. According to this study, Gal-3 null animals are more prone to develop greater liver steatosis but there is decreased inflammation, liver cell injury and fibrosis. Since Gal-3 appears to be a critical regulator of liver fibrosis attempts have been made to inhibit its activity by means of a specific inhibitor, named GR-MB-02, in mice [337], in rats [338] and in humans [339]. In particular, the human study was a sequential dose-ranging, placebo controlled, double-blinded study and was undertaken to evaluate the safety, pharmacokinetics profiles and to evaluate changes in potential serum biomarkers and liver stiffness as assessed by FibroScan in subjects having NASH with bridging fibrosis. GR-MD-02 proved to be safe and well tolerated with evidence of a pharmacodynamic effect. Such results constitute the basis for a Phase 2 development program of treatment with GR-MD-02 in advanced fibrosis due to NASH. At the present time, the smaller four-month long phase 2a trial, involving a small set of 30 NASH patients with advanced fibrosis (NASH-FX) at a single site, indicate that the results missed the primary goal and a few secondary endpoints [340], but additional positive results are expected from the larger in-progress phase 2b clinical trial (NASH-FX) [341].

## 17. O

### Obesity

Obesity is a common risk factor for metabolic syndrome, a group of several diseases that include insulin resistance, hypertension, glucose intolerance and toxicity, hepatic steatosis, atherogenic dyslipidemia, and T2DM [342]. Obesity correlates with chronic, low-grade inflammation, which appears to be necessary for obesity-associated insulin resistance and the subsequent development of T2DM [343]. During the development of obesity, macrophages permeate white adipose tissue and, together with adipocytes, secrete various pro-inflammatory cytokines and chemokines [344,345]. In obese individuals the visceral adipose tissue is infiltrated by macrophages that cooperate in generating and sustaining the inflammatory responses [346]. Gal-3 is expressed in all types of macrophages that permeate white adipose tissue and it is an important regulator of both polarization and activity, thus mediating both pro-inflammatory and anti-inflammatory effects in such cells [347,348]. Experimental evidence, using Gal-3^−/−^ mice indicate that in vivo Gal-3 administration causes glucose intolerance and insulin resistance whereas in vitro treatment with selective small molecule Gal-3 inhibitors can directly induce decreased insulin sensitivity in myocytes, hepatocytes, and adipocytes [349]. Such experiments suggest a possible interesting link between inflammation and decreased insulin sensitivity.

## 18. P

### 18.1. Pneumonia

The Gram-positive bacterium *Streptococcus pneumoniae* is the most common cause of community-acquired pneumonia, still responsible of a high mortality rate, especially in children and in developing Countries [350]. In the lung, resident alveolar macrophages are the first line of cellular defense and play a phagocytic role during the early stages of infection. Gal-3 is abundantly expressed and secreted by macrophages [298] and clearance of apoptotic neutrophils by macrophages is a key step in the resolution of inflammation. Using an in vivo pneumonia mouse model in which Gal-3 was knocked out, it has been demonstrated that Gal-3 is involved in the recruitment of neutrophils during lung infections with *Streptococcus pneumoniae* [301]. In addition, it was demonstrated that the absence of Gal-3 is responsible for an inadequate host response to pneumococcal infection, resulting in high mortality of the Gal-3-null mice [74,351,352]. In agreement with this observation, the exogenous administration of Gal-3 was able to enhance phagocytosis of *Streptococcus pneumoniae* by both WT and Gal-3^−/−^ neutrophils [74,351]. In the same studies it has been demonstrated that administration of exogenous Gal-3 had a direct antimicrobial, bacteriostatic effect on *Streptococcus pneumoniae*. Gal-3, in fact, is able to inhibit *in vitro* growth of *Streptococcus pneumoniae* and to reduce the severity of bacteremia and of the pneumonia in vivo. These very interesting and promising effects of Gal-3 may have relevant clinical implications in the treatment of *Pneumococcal pneumonia* in humans but needs to be further investigated.

### 18.2. Pulmonary Hypertension

Pulmonary arterial hypertension (PAH) is a heterogeneous group of diseases. It includes the idiopathic pulmonary hypertension characterized by a persistent increase in pulmonary arterial resistance often related to small pulmonary arterial vessel remodeling and endothelial metabolic alteration with consequent deterioration of lung and heart functions. During PAH increase of pulmonary pressure is determined by microvascular inflammation, endothelial dysfunction, activation of vascular smooth muscle cells and vascular thrombosis [353]. In this context, Gal-3 is able to modulate fibroblast and endothelial behavior acting on fibroblast and vascular endothelial growth factors [354]. Currently, there are few and contrasting studies addressing the precise role of Gal-3 in PAH development and progression. This confusion may be due to the different PAH patient characteristics like the presence of comorbidities or diseases secondary complicated by PAH like connective tissue diseases, respiratory or hearth diseases. Song et al. reported in 2016 that Gal-3 plasma levels are decreased in patients with PAH in comparison with healthy controls [355]. Moreover, Gal-3 mRNA was down-regulated in the pulmonary vessels in pulmonary hypertensive rats [355]. Contrarily, elevated level of Gal-3 has been reported in PAH independently to etiology and associated with severity of PAH [356,357]. Gal-3 plasma levels are correlated with right ventricle dysfunction [358] and with extracellular matrix markers [356,357]. Gal-3 seems to play a key role in acute and chronic inflammatory responses to endothelial injury, vascular remodeling and fibrosis in PAH [359]. The serial measurement of Gal-3 serum levels was suggested as a useful marker to improve prediction and progression of PAH [360]. A preclinical study demonstrated that Gal-3 might be also useful as a target for the treatment of this condition. In fact, the treatment with Gal-3 inhibitors was able to counteract lung and heart remodeling and fibrosis in a rat model of PAH [361].

### 18.3. Plaque Psoriasis

Gal-3 is abundantly expressed in the cytoplasm of normal keratinocytes, in hair follicles, sebaceous, and sweat glands [87]. Moreover, Gal-3 is associated with keratinocyte differentiation and maturation [362].

Plaque psoriasis is one of the most prevalent autoimmune skin diseases. It is characterized by the presence of well-demarcated red silvery scales on the skin surface. Gal-3 was expressed in a high proportion of Langerhans cells from skin punch biopsies, obtained from lesional plaque-type psoriatic skin [363]. In addition, a strong expression of Gal-3/Gal-3-reactive glycoligands was detected in capillaries of psoriatic dermis [364].

However, the most relevant indication regarding the role of Gal-3 in psoriasis was obtained unexpectedly during the exploratory phase 2 clinical trials on the use of GR-MD-02 in nonalcoholic steatohepatitis (“NASH”) (ClinicalTrials.gov Identifier: NCT01899859). A side effect of the treatment was, in fact, the evidence of long-term remission or significant improvements in disease activity of psoriasis in patients treated with GR-MD-02. This finding suggested that blocking Gal-3 could be effective in treating moderate to severe plaque psoriasis patients. That is why a new single-center, open-label phase 2a clinical trial was initiated to evaluate safety and efficacy of GR-MD-02 for treatment of psoriasis (ClinicalTrials.gov Identifier: NCT02407041).

## 19. Q

### Q Fever

The etiologic agent of Q-fever is a highly infectious human pathogen named *Coxiella burnetii* [365]. *Coxiella burnetii* is an obligate intracellular bacterium that inhabits mainly monocytes/macrophages. It is known that galectins are recruited to injured membranes by their interactions with N-glycans that are exposed to the cytoplasm when the membrane of a vesicle is pierced and, therefore, are considered useful molecules to detect damage of intracellular membranous compartments. In this regards, there is evidence derived from infection by two bacterial pathogens, namely the *Shigella flexneri*, a rod-shaped, Gram-negative bacterium responsible for bacillary dysentary and the *Listeria monocytogenes*, a rod-shaped Garm-positive bacterium that causes severe meningitis and bacteriemia in immunocompromised hosts, that demonstrated that Gal-3 accumulates in structures in close proximity to phagosomes containing bacterial pathogens and are useful to detect membrane damage in bacteria’s vacuoles [366,367,368]. These bacteria share similar lifestyles in the sense that by producing surface proteins they both lyse their phagosome and induce their own phagocytosis by host cells [369]. In such bacteria it has been postulated that Gal-3 may function as a danger receptor for membrane damages [366,367]. Similar to the behavior of these two pathogens also *Coxiella burnetii* was found to transits through the endocytic pathway, which delivers the bacteria to the low pH environment of a lysosome. Several members of the galectin family interact with the *Coxiella burnetii* containing vacuole, at different times of infection. The role of the expression of Galectins, and in particular of Gal-3 in the lysosomes of the infected cells appears to be crucial. It is believed that when lysosomes are damaged they are marked with Gal-3 and, as a consequence, can be targeted to autophagy [370]. This study points to Gal-3 as a sensor of damage at the *Coxiella burnetii* limiting membrane and indicates a possible role of Gal-3 in the mechanisms of autophagy during membrane repair.

## 20. R

### 20.1. Rheumatoid Arthritis

In contrast to Gal-1 that act as a negative regulator of autoimmunity in rheumatoid arthritis (RA), Gal-3 promotes inflammation in RA. The role of Gal-3 as a pro-inflammatory mediator in RA is based on several lines of evidence, both in animals and in humans [371].

#### 20.1.1. Experimental Studies in Animals

Studies with collagen-induced arthritic (CIA) rats found increased Gal-3 secretion into the plasma over time, which correlated with the disease progression, implicating that Gal-3 promotes the development of experimental arthritis [372]. Recent studies with Gal-3-deficient mice further confirmed the stimulating role of Gal-3 in arthritis [373]. The joint inflammation and bone erosion of antigen-induced arthritis were markedly suppressed in Gal-3-deficient mice as compared with the wild type mice. The reduced arthritis in Gal-3-deficient mice was accompanied by a decreased level of antigen-specific IgG and pro-inflammatory cytokines, including TNFα, IL-6, and IL-17. Furthermore, an exogenous supply of recombinant Gal-3 restored the reduced arthritis and cytokine production in Gal-3-deficient mice [373]. This study provided the direct evidence that Gal-3 plays a crucial role in the development of arthritis in animal models.

#### 20.1.2. Clinical Studies in Humans

The role of Gal-3 in RA was also demonstrated in humans where Gal-3 was found to be increased in the serum and synovial fluid of RA patients with long-standing disease, compared with osteoarthritis (OA) as well as in JIA patients [374,375,376]. The serum levels of Gal-3 were elevated in patients with RA, JIA, Behçet’s disease, or systemic sclerosis [250,315,374,377]. Although the increased Gal-3 is not specific for RA, the serum levels of Gal-3 were significantly associated with the C-reactive protein (CRP) levels and the disease activity scores in patients with JIA, suggesting that Gal-3 may be utilized as a biomarker for the disease progression of JIA [315]. In addition, the *LGALS3* gene allele (*LGALS3* + *292C*) is more prevalent in RA patients than in healthy controls, indicating that genetic polymorphisms of Gal-3 may influence the susceptibility to RA [378]. In addition to immune cells, fibroblast-like synoviocytes (FLS) in the synovium of RA patients also express Gal-3 at high levels [379,380,381]. While floating FLS only express low levels of Gal-3 [379], adhesion of FLS to cartilage components through CD51/CD61 induces Gal-3 expression [375]. In RA patients, about 39% of FLS are cartilage-adhering cells, which is four times more than in osteoarthritis (OA) patients. The increased numbers of adhering FLS contribute to the elevated Gal-3 levels in the RA synovium [375]. Moreover, Gal-3 can induce rheumatoid FLS to secret a set of pro-inflammatory cytokines and chemokines including IL-6, granulocyte-macrophage colony-stimulating factor (GMCSF), TNF, CXCL8, CCL2, CCL3, and CCL5 [381]. The induction of cytokines and chemokines by Gal-3 appears to involve different signaling pathways. The MAPK-ERK pathway was necessary for cyotokine IL-6 production, while phosphatidylinositol 3-kinase (PI3K) was required for chemokine CCL5 induction [381]. These studies using human materials further suggest a promotional role of Gal-3 in the pathogenesis of RA. In addition, Gal-3 mRNA and Gal-3 binding protein were highly expressed in RA joints, particularly at the sites of cartilage and bone destruction [377]. Gal-3 was reported to contribute to the activation of RA synovial fibroblasts [377] and to promote the production of pro-inflammatory cytokines, such as interleukin-6 and tumor necrosis factor-α, in synovial fibroblasts [381,382]. For all these reasons the Gal-3 serum level was proposed as a predictive marker of future RA [383]. According to this study, the increase of serum levels of Gal-3 may help in discriminating between patients with undifferentiated arthritis who subsequently developed RA and those with undifferentiated arthritis who do not develop RA and in this sense it works better than other serological markers of cartilage regeneration (such as the type IIA collagen N-terminal propeptide, PIIANP) or synovial swellings (such as the hyaluronan, HYA). The therapeutic administration of lentiviral vectors encoding Gal-3 small hairpin RNA (shRNA), directly injected intra-articularly, into the ankle joints of rats with collagen-induced arthritis, significantly ameliorates the disease activity [384]. This treatment, in fact, reduced articular index scores, radiographic scores and histological scores and was accompanied with decreased T-cell infiltrates and reduced microvessel density in the ankle joints. All these experimental findings indicate that Gal-3 emerges not only as a key player in the pathogenesis of RA [385], but also as a novel potential therapeutic target and implicate that down-regulation of Gal-3 may represent novel a therapeutic strategy for RA.

## 21. S

### 21.1. Sepsis

Sepsis is a complex immune disorder with a high mortality rate of 20–50% that currently has no therapeutic interventions [386]. There are few studies that suggested a possible role of Gal-3 in inflammatory conditions, including endotoxemia and airway inflammation [71,387,388].

The relationship between Gal-3 and sepsis was recently studied using a murine inhalation model, infected with *Francisella novicida*, a Gram negative bacterial pathogen, responsible for the development of severe sepsis characterized by hyperinflammation, T cell depletion, and extensive cell death in systemic organs. The measurement of both mRNA and protein levels of Gal-3 in lungs of mice undergoing lethal pulmonary infection with such pathogen, indicated a significant increase in Gal-3 expression [386]. This increase was evident 3 days after lethal injection and was associated with the appearance of other sepsis features, such as extensive cell death, hyperinflammatory response and increased vascular injury. When the same treatment was given to Gal-3^−/−^ mice, a significant reduction in levels of several vascular injury markers and of several inflammatory cytokines was observed. In addition, Gal-3^−/−^ mice showed significantly improved survival as compared to the infected wild-type mice. These results indicate a strong immune-stimulatory role of Gal-3 during pulmonary lethal infection and indicate that Gal-3 may represent a potential target for treatment of sepsis, at least for that induced by *Francisella novicida*.

However, the situation may be different when other pathogens are involved. In fact, lipopolysaccharide expressed on *Escherichia coli* has been shown to down-regulate Gal-3 expression [298,389].

Results similar to those observed with *Francisella novicida* were obtained also in the case of infection with *Streptococcus pneumonia*. In this regard, it has been demonstrated that, after pneumococcal infection of the lungs, Gal-3 accumulates in the alveolar space and this accumulation correlates with the onset of neutrophil activation [301]. Infection of mice with the Gram-positive *Streptococcus pneumoniae* was more severe when it was induced in Gal-3^−/−^ mice, compared to wild-type, and Gal-3 released from resident alveolar macrophages enhance their bacterial killing and phagocytic capability of neutrophils, aiding pathogen clearance [351]. These data suggest that, conversely to what reported in sepsis induced by *Francisella novicida*, Gal-3 may play a protective role against sepsis due to pulmonary infection by *Streptococcus pneumoniae*. Recently, the hypothesis that Gal-3 could be useful as a marker to predict mortality was tested in a group of 273 patients affected by sepsis and analyzed retrospectively [390]. Gal-3 was measured in association with other biomarkers, such as procalcitonin, presepsin, and soluble ST2. Gal-3 showed the strongest prognostic value in predicting mortality in septic patients. Further studies are required to elucidate the real diagnostic and prognostic usefulness of Gal-3 in septic patients as well as its potential therapeutic use.

### 21.2. Systemic Sclerosis

Systemic sclerosis (SSc), also known as scleroderma, is a connective tissue disease characterized by vasculopathy and progressive fibrosis of the skin and certain internal organs, due to activation of fibroblasts and progressive accumulation of extracellular matrix molecules, eventually leading to internal organ dysfunction [391]. Considering the known relevant role of Gal-3 in fibrosis [333] it is obvious that it was analyzed in a disease such as SSc in which progressive fibrosis of the skin and of visceral organs represents a major component of the disease. Serum Gal-3 levels were, in fact, analyzed in patients affected by SSc in some studies. They were found to be significantly decreased in early diffuse cutaneous SSc, compared with the control subjects, but showed increase during the course of the disease [250]. In addition, in diffuse cutaneous SSc, a significant correlation was reported between serum Gal-3 levels and the developmental process of skin sclerosis and of digital ulcers [250]. In another study, serum Gal-3 levels were found to be higher in SSc patients than in healthy controls [251]. In order to evaluate the possible associations between Gal-3 levels and patient characteristics and to investigate its long-term prognostic value, a large population of 152 patients that fulfilled the American College of Rheumatology criteria for SSc [392] was recently analyzed [255]. In multivariate Cox regression analyses and in a long-term follow-up (7.2 ± 2.3 years), Gal-3 was an independent predictor both of the all-cause and of cardiovascular-related mortality, even after adjustment for age, gender, BSA, creatinine and NT-proBNP levels. In particular, patients with elevated serum Gal-3 levels (>10.25 ng/mL) showed a clear association with the signs of the advanced organ sclerosis and with the laboratory parameters of inflammation. Gal-3 appears to be a reliable biomarker to predict all-cause mortality in patients affected by SSc.

## 22. T

### Target Therapy

Gal-3 is involved in the regulations of a myriad of cell activities. As a multifunctional protein widely expressed by many types of human cells, it interacts with a range of different binding partners at different locations. In addition, its overexpression in many different conditions and changes of its sub- and inter-cellular localization are commonly seen in various types of human diseases. Gal-3 research is one of the fastest growing fields within medicine today and Gal-3 is becoming the most informative new biomarker for our most serious health epidemics for which early detection is critical to successful clinical prognosis. A growing body of evidence demonstrates, in fact, how Gal-3 is involved in numerous degenerative processes within the body, most notably cancer proliferation/metastasis, heart failure, atherosclerosis, diabetes, chronic inflammation, fibrosis and related organ failure. In addition, Gal-3 was found to be a critical prognostic factor even in all-cause mortality. Therefore, it is not surprising that Gal-3 represents an attractive molecule to be targeted for the development of new strategies in the treatment of many different diseases [64]. The studies concerning the possibility to inhibit Gal-3 function were driven by the experiments using Gal-3 null mice. Basically, two different approaches were taken in developing drugs that target the Gal-3 molecule. One approach is based on the use of modified disaccharides. One example of such compound is the TD139 (Galecto Biotech). Another approach is to use naturally occurred large polysaccharides that contain galactose. Example of such naturally occurred large polysaccharides are the modified citrus pectins (MCP), and in particular: The GR-MD-02, belonging to the galacto-rhamnogalacturonate (GR) class and the GM-CT-01, belonging to the galactomannan (GM) class, by Galectin therapeutics Inc. (Norcross, GA, USA) [393]. Recently, another MCP compound, named PectaSol-C, was introduced and commercialized by EcoNugenics (Santa Rosa, CA, USA). It was demonstrated to be able to reduce Gal-3 serum levels in vivo in both mice [394] and humans [395].

The Gal-3 antagonist named GCS-100 is a MCP, capable of binding to and antagonizing Gal-3, developed (La Jolla Pharmaceutical Company, San Diego, CA, USA) and utilized in a clinical trial (ClinicalTrials.gov Identifier: NCT00776802) for the treatment of refractory diffuse large B-cell lymphoma and refractory solid tumors [396]. The trial was discontinued in 2015 because of lack of funding. The same drug was used in another interventional trial (ClinicalTrials.gov Identifier: NCT01843790) for the treatment of patients with chronic kidney disease. Pectins are available as various types of dietary fibers (e.g., fruit, vegetables, sugar beets). They constitute the fibers found in the pith (the bitter, white stuff) in the peels of fruit, particularly rich in oranges, grapefruits, and lemons. Pectins are commonly used as a thickening agent in jams and baked goods. Pectins have been identified as Gal-3 inhibitors. Both pectins and MCP bind to the CRD of Gal-3 and neutralize its activity [397]. They were reported to be able to inhibit cancer growth and to be an effective anti-fibrotic agent in mouse models [337,338,398,399,400].

A large numbers of clinical trials are now ongoing for many different clinical conditions. Currently, there are 9 interventional clinical trials ongoing regarding the therapeutic use of Gal-3 inhibitors, registered at the web site*ClinicalTrials.gov*, maintained by the National Library of Medicine (NLM) at the National Institutes of Health (NIH) [401]. They include chronic inflammation and fibrosis affecting the lung (ClinicalTrials.gov Identifier: NCT02257177) and the kidney (ClinicalTrials.gov Identifier: NCT01717248 and NCT01843790), cirrhosis and portal hypertension (ClinicalTrials.gov Identifier: NCT02462967), high blood pressure (ClinicalTrials.gov Identifier: NCT01960946), osteoarthritis (ClinicalTrials.gov Identifier: NCT02800629), and combination immunotherapy for cancer, including melanoma (ClinicalTrials.gov Identifier: NCT02117362 and NCT01723813), large B-cell lymphoma (NCT00776802, withdrawn prior to enrollment for lack of funding). In addition, the occurrence of serendipity has led to expand the potential clinical use of Gal-3 inhibitors also to skin diseases. In fact, administration of GR-MD-02 to a patient enrolled in a Phase I NASH trial induced a remarkable remission of psoriasis, indicating the potential importance of Gal-3 in such disease. Following this observation, an open-label, phase 2a study was started in 2015 to evaluate safety and efficacy of GR-MD-02 for the treatment of psoriasis (ClinicalTrials.gov Identifier: NCT02407041). One could argue that a new science is born, called “Pectin Science” or “Pectinology” [400]. The analysis of health promoting capacities of Pectins is progressing fast and will provide interesting results in the near future.

## 23. U

### Urinary Tract Infections

Various galectins have been shown to bind a wide range of pathogens, such as Gram-positive bacteria (e.g., *Streptococcus pneumoniae*), Gram-negative bacteria (e.g., *Klebsiella pneumoniae*, *Neisseria meningitidis*, *Neisseria gonorrhoeae*, *Haemophilus influenzae*, *Pseudomonas aeruginosa*, *Porphyromonas gingivalis*, *Helicobacter pylori* and *Escherichia coli*), enveloped viruses (*Nipah* and *Hendra* paramyxoviruses, *human immunodeficiency virus-1, HIV-1*, and *influenza virus A*), retrovirus (*human T-lymphotropic virus, HTLV-1*), hantavirus, fungi (*Candida albicans*) and parasites (*Toxoplasma gondii*, *Leishmania major*, *Schistosoma mansoni*, *Trypanosoma cruzi*, and *Trichomonas vaginalis*) [259,282,402,403,404,405,406,407,408,409,410]. The specific role of Gal-3 in urinary tract infections in humans was analyzed in case of viral and bacterial infections. Gal-3 serum levels were increased in patients with chronic viral infectious disease such as hepatitis C [411] and HIV [412]. In particular, an elevated Gal-3 serum level was associated with unresponsiveness to alpha-interferon treatment and a strong association was observed between viral load and Gal-3 expression in HIV-infected patients. Gal-3 was also investigated in case of urinary tract infection due to bacterial infection. Gal-3 was found to play a crucial role in bacterial infections, and in particular in infections due to *Proteus mirabilis*, a bacterium frequently detected in the urinary tract. Gal-3 influences adhesion of such bacterium to the extracellular surface of the plasma membrane of different kidney epithelial cell lines and its crucial role in *Proteus mirabilis* infection was demonstrated using specific monoclonal antibodies directed against Gal-3 [413]. These studies indicate that Gal-3 plays a key role of in renal pathology due to infections [414].

## 24. V

### Venous Thrombosis

Gal-3 is associated with thrombogenesis and in particular, it is characterized by prothrombotic and pro-inflammatory properties in the context of experimental venous thrombosis. Serum levels of Gal-3 and of Gal-3 binding protein are elevated in the course of venous thrombosis in both mice and humans [415]. Circulating Gal-3 is particularly elevated during the early phases of venous thrombosis and its level correlates with the size of the thrombus. Gal-3 appears to be increased both locally, in the thrombus and in the vein wall, and systemically, in blood-circulating elements, such as red blood cells, with blood cells, platelets and microparticles. Therefore, Gal-3 may represent an ideal target for therapies aimed to prevent venous thrombosis/pulmonary embolism [415].

## 25. W

### 25.1. Wound Healing

Many studies, performed in epithelium form different tissues, indicate that one of the best-known functions of Gal-3 is to promote cell migration and wound re-epithelialization [416,417,418,419]. Impaired wound healing and healing defects may affect the epithelium in many different organs systems, including the skin, the gastrointestinal tract and the corneal epithelium [420,421,422,423,424]. In addition, the damage of delayed re-epithelialization and the occurrence of persistent epithelial defects are also observed in chronic wounds of the elderly, decubitus ulcers as well as in venous statis ulcers of the skin. The major defect responsible for the failure to re-epithelialize in generally thought to be the reduced ability of the epithelial cells to migrate across the wound healing bed more than a defect in cell proliferation [425] and Gal-3 is able to modulate cell migration through its effects on cell adhesion and its interactions with cell-matrix. For these reasons Gal-3 is considered a key player in the re-epithelialization of wounds in many different tissues [426].

#### 25.1.1. Wound Healing in the Cornea

The effects of Gal-3 in the process of corneal re-epithelialization of wounds have been analyzed using a Gal-3 null mouse model [416]. In such mouse model, the absence of Gal-3 impairs keratinocyte migration and skin wound re-epithelialization. Using a mouse model in which corneas were injured using both laser or alkali treatment it has been demonstrated that corneal epithelial wound closure rate was significantly slower in Gal3^−/−^ mice compared with wild-type mice [416]. Since the rate of corneal epithelial cell proliferation is not altered in Gal3^−/−^ mice, it is likely that the effect is due to impairment in the cell migration process. To confirm this observation the mouse corneas were incubated in in serum-free media, in the presence of different amounts of recombinant Gal-3 protein. Exogenous Gal-3 was able to stimulate the rate of wound closure in a concentration-dependent manner [416]. The same effect was demonstrated the corneal epithelium of rats [427] and monkeys [428].

#### 25.1.2. Wound Healing in the Skin

It is known that the EGF-stimulated EGFR-ERK signaling is essential for keratinocyte migration, a crucial step for skin wound re-epithelialization [429,430,431]. The role of Gal-3 in skin wound re-epithelialization was analyzed in a Gal-3^−/−^ mouse model [432]. Keratinocyte migration is impaired in Gal-3^−/−^ mice. Gal-3 exerts this effect by regulating the intracellular trafficking of EGFR.

#### 25.1.3. Wound Healing in the Intestinal Tract

Using the in vitro model of T84 human colonic adenocarcinoma cell line, it was demonstrated that the Gal-3 promotes wound healing and that the cleavage of Gal-3 by Matrilysin-1 (MMP7) results in the overall abrogation of such wound healing capacity [433]. Based on this observation it has been hypothesized that cleavage of Gal-3 is one mechanism by which MMP7 inhibits wound healing. The relevance of such finding and its possible clinical role in conditions such as intestinal ulcers and chronic inflammatory bowel diseases still needs to be elucidated.

## 26. X

### X Syndrome of the Heart 

The X syndrome of the heart, also worded as cardiac syndrome X (CSX) and differing from the other X syndrome of the metabolism, also known as metabolic syndrome X (MSX), refers to the triad of typical effort angina, positive stress test and normal epicardial coronary angiogram [434]. It can be diagnosed in about 3–11% of patients undergoing coronary angiography because of typical chest pain [435]. Follow-up studies of patients with CSX generally report good prognosis [436,437]. A systematic review of the literature shows that the CSX prognosis in terms of the overall cardiac event rate is excellent, with a risk of myocardial infarction or cardiovascular death of 1.5% per five years [438]. However, even if such patients have been considered to be a low-risk population the occurrence of cardiovascular events in such patients is not infrequent, especially in women [439]. Many attempts have been made to find useful markers for identifying the CSX patients at high risk of cardiovascular disease, and CRP, interleukin-6 and uric acid as well as the occurrence of perivascular fibrosis, with higher carotid mean intima-media thickness, and apoptosis of endothelial cells in the small blood vessels were found to be associated with such syndrome [440,441,442]. Serum levels of Gal-3 were measured in a cohort of 115 patients affected by CSX demonstrated. They were significantly higher in the CSX patients in comparison with the healthy controls [443]. This preliminary study needs to be validated in larger number of patients. However, it suggests that Gal-3 may play a key role in the CSX and may represent a useful marker for identifying those CSX patients that are at high risk of developing cardiovascular disease.

## 27. Y

### Yeast Infection-Candidiasis

Galectin family of lectins participates in the innate immune defense against pathogens [404]. Gal-3 was shown to have antimicrobial activity toward the pathogenic fungus *Candida albicans* [302]. Candida species are the fourth leading cause of nosocomial bloodstream infections. They are responsible for life threatening disease in immunocompromised individuals. Invasive candidiasis affects more than 250,000 people each year and leads to more than 50,000 deaths worldwide. In the United States mortality due to systemic, invasive infections by *Candida albicans* can be as high as 40% even after receiving antifungal therapy [444,445,446]. Vulvovaginal candidiasis affects 75% of women of childbearing age [447]. It is interesting to note that Gal-3 is normally expressed by epithelia lining the body surfaces that are exposed to *Candida* colonization, such as vulvar and oral mucosa, corneal, conjunctival and intestinal epithelia [448,449,450,451]. Gal-3 appears to be present in the fungal granulomata, it binds to *Candida albicans* in a carbohydrate-specific manner and it displays a direct fungicidal activity that may be restricted to specific Candida species. This is the first observation regarding the direct antifungicidal activity for a member of the galectin family of mammalian lectins. The ability to induce Candida death is a unique function of Gal-3, because galectin-1 is not able to bind to any Candida species examined. More recently Gal-3, secreted from neutrophils, was also demonstrated to be also active against other *non-albicans* species, such as the *Candida parapsilosis* and the *Candida albicans hyphae* [452]. Gal-3, in fact, acts as a pro-inflammatory autocrine/paracrine signal in neutrophil phagocytosis. This observation suggests that Gal-3 has a unique role in neutrophil response to *Candida parapsilosis* yeast and *Candida albicans hyphae* distinct from *Candida albicans* yeast. In addition, Gal-3 plays an important role in host recognition and response to candida infection, as demonstrated using a murine model of disseminated candidiasis [453]. In such murine model, knockout of the Gal-3 gene (Gal-3^−/−^ mice) conferred susceptibility to *Candida* infection compared to wild type ones (Gal-3 WT-mice) [453]. When infected with the less virulent specie, namely the *Candida parapsilosis*, Gal-3^−/−^ mice had significantly higher renal fungal burdens and abscess formation compared to Gal-3 WT-mice. This study suggests a potential mechanism of neonatal susceptibility to these infections.

Additional informations will be obtained from studies aimed to analyze the gene polymorphisms of candidate genes associated with yeast colonization [454,455]. In this regard, a new clinical trial is now ongoing to analyze the innate immunity gene polymorphisms of the so-called “Candigene”. In particular, aim of the study will be the genotyping of the following lectins: Mannose-binding lectin (MBL), the Dectin-1 and the Gal-3 (ClinicalTrials.gov Identifier: NCT02888860).

The mechanism of Gal-3 secretion by the neutrophils and how it influences host defense was very recently investigated [456]. According to this study Gal-3 deficiency ameliorates systemic candidiasis by reducing fungal burden, renal pathology, and mortality. These studies indicate that blockade of Gal-3 in neutrophils may be a promising therapeutic strategy for systemic *Candida* infection. The inhibition of Gal-3, obtained by knocking down the gene, by silencing its expression or by administration of inhibitors such as the newly identified and TD139 molecule [457], patented by Galecto Biotech [458], is able to inhibit neutrophil reactive oxygen species (ROS) production and enhance the ability of neutrophils to kill when encountering “hard-to-kill” Candida.

## 28. Z

### Zoster-Related Pain (Allodynia)

*Herpes zoster* (HZ) is caused by the reactivation of *varicella zoster virus* (VZV) after being latent in the sensory ganglia [459,460] and is characterized by blistering skin eruption and neuropathic pain in the affected dermatome [461]. It has been calculated that approximately 30% of population will suffer of HZ during their lifetime [462]. Persistence of pain that lasts after the rash and blisters have healed, a condition known as postherpetic neuralgia (PHN) or zoster-related pain, is considered as the most common and severe complication of HZ [463]. The sensory changes within the affected dermatome, associated with both HZ and PHN, may induce reduced sensitivity of the skin as well as allodynia, that is occurrence of pain due to stimulus which does not normally provoke pain [464]. Gal-3 is highly expressed in peripheral nervous tissues of adult rodents, where is localized to cell bodies of sensory neurons, axons and Schwann cells [465] and in the autonomic preganglionic neurons in the spinal cord [466]. There is growing evidence suggesting that Gal-3 is involved in the process of Wallerian degeneration distal to the injury and subsequent axonal regrowth and functional re-innervation [221]. To identify the genes involved in acute herpetic pain and analyze the role of Gal-3, a mouse model (C57BL/6J) was subjected to transdermal *Herpes simplex virus-1* (HSV-1) inoculation in the hind paw, to produce *Herpes zoster*-like skin lesions and mechanical allodynia. In such mouse model Gal-3 expression levels as well as the global gene expression profiles of the dorsal cord, at the L4 to L6 levels, were analyzed by GeneChip oligonucleotide expression arrays [236]. Gal-3 mRNA levels were slightly induced on day 5 after inoculation, rapidly increased from day six to day eight, and then gradually decreased. HSV-1 induced mechanical allodynia (day six after inoculation) caused a strong induction of Gal-3 protein expression, analyzed by immunohistochemical methods, in the superficial part and sparsely in the deep part of the lumbar dorsal horn on the HSV-1-inoculated side. To test whether Gal-3 contributes to herpetic allodynia, the effects of deficiency in the Gal-3 gene were analyzed using Gal-3^−/−^ mice. In such mice, the onset (day five) and peak (around day seven) of HSV-1 induced allodynia were similar to those of wild-type mice, but the intensity of the pain was significantly reduced. *Zoster*-like skin lesions were not affected by the Gal-3 gene deficiency. Finally, the intrathecal injection of rat and goat anti-Gal-3 antibodies significantly ameliorated HSV-1-induced allodynia. Considering all these observations is not surprising that Gal-3 was considered as a key molecule in the pathogenesis of neuropathic pain following peripheral nerve injury and tested for its possible therapeutic use. In a rat model (Sprague–Dawley) in which neuroinflammation and neuropathic pain were induced by L5 spinal nerve ligation (SNL), an increase in the mRNA and protein expression levels of Gal-3 was observed in dorsal root ganglions [467]. Intrathecal administration of modified citrus pectin (MCP), a Gal-3 inhibitor, was able to inhibit such effect, causing a reduction of Gal-3 expression in dorsal root ganglions. MCP treatment also inhibits SNL-induced Gal-3 expression in primary rat microglia and decreases LPS-induced expression of pro-inflammatory mediators, such as IL-1β, TNF-α and IL-6. Gal-3 inhibition resulted in a significant attenuation of neurophathic pain and in a decreased mechanical and cold hypersensitivity. Gal-3 emerged as a new target for the treatment and clinical management of peripheral nerve injury-induced neuropathic pain. No clinical studies regarding the potential role of Gal-3 in postherpetic neuralgia in humans have been published yet.

## 29. Conclusions

This review represents an attempt to describe all the known clinical conditions in which a role of Gal-3 was postulated or demonstrated. All the various clinical diseases and conditions, reported in this review in alphabetical order, in which Gal-3 was analyzed and for which a specific clinical pathogenic, diagnostic or prognostic role of Gal-3 was proposed are summarized in Table 1. However, this list should not be considered completed yet. It is likely that many other diseases are still waiting to be analyzed and added to it. It is impressive to realize how relevant is the clinical impact of all such studies on Gal-3 and how fast was the introduction of clinical tests based on Gal-3 into clinical practice to be used as diagnostic or prognostic biomarkers in many different clinical contexts. Even much impressive is the prompt use of all the information regarding the pathogenic role of Gal-3 in severe diseases where few or no therapeutic options are available to design new target treatments focused on Gal-3. Many different clinical trials targeting Gal-3 molecule are still ongoing to treat severe neoplastic, fibrotic, metabolic and degenerative disease and many pharmaceutical companies are investing money and efforts to develop and test drugs targeting the Gal-3 molecule and acting as antagonist or agonist. The astonishing clinical history of this extraordinary lectin and the new science called “Pectin Science” or “Pectinology” are just at the very earl stages and the dawning of a new era with new exciting evolutions are expected soon.

## Figures and Tables

**Table 1:** Conditions and diseases, listed in alphabetical order, in which a possible role of Gal-3 was postulated in pathogenesis, diagnosis, prognosis, stratification for therapy and in therapy.

Letter Names	Disease/Condition	Possible Role in Pathogenesis	Possible Role in Diagnosis/Prognosis	Possible Role in Patients Stratification for Therapy	Possible Role in Therapy
A	Asthma			Gal-3 positive patients are more responsive to omalizumab	
	Atherosclerosis	Gal-3 is expressed in advanced atherosclerotic lesions			
	Atopic Dermatitis	Gal-3 released by PMNs amplifies IgE production			Gal-3 inhibitors used in a phase 2 controlled trial for treatment of severe atopic dermatitis
B	Blood test		Blood assays (ELISA) to predict outcome of acute and chronic heart failure		
C	Cancer	Gal-3 controls oncogenesis, cancer progression and metastasis	Diagnostic role in thyroid, prostate, and ovarian cancers		Clinical trials using Gal-3 inhibitors for melanoma and large B-cell lymphoma
	Cerebral infarction	Serum levels of Gal-3 are increased in ischemic brain damage	Measurement of serum Gal-3 levels may help in diagnosis of cerebral infarction		
	COPD	Gal-3 levels measured in BAL are reduced in healthy smokers	In patients with COPD serum Gal-3 levels may predict right ventricular dysfunction and acute exacerbation of COPD		
D	Degenerative Aortic Stenosis			Serum Gal-3 levels may be useful in stratification for therapy in elderly patients affected by degenerative aortic stenosis	
	Diabetes Mellitus	Conflicting results: Gal-3 plays a rotective role; Gal-3 is a risk factor for vascular/renal/cardiac complications			
E	Endometriosis	Gal-3 is overexpressed in endometriotic tissue			
	Enteric nervous system	Gal-3 is crucial mediator of the GI damage observed after brain injuries, at the level of enteric neuronal cells			
	Encephalitis	Gal-3 is involved in various types of CNS inflammation and in particular in viral encephalitis			
F	Fibrosis	Gal-3 is key regulator of chronic inflammation and tissue fibrogenesis	Serum Gal-3 levels are increased in fibrosis affecting the liver, the kidney, the lung, the heart, the nervous system and in the systemic sclerosis. In such conditions Gal-3 has been proposed as a new prognostic factor		
G	Gastritis	Gal-3 is upregulated in gastric epithelial cells following adhesion of *Helicobacter pylori*			Experimental evidence indicates that administration of extracellular recombinant Gal-3 (rGal-3) is able to inhibit the adhesion of *Helicobacter pylori* to the gastric epithelial cells
H	Heart		Serum Gal-3 levels are associated with mortality in patients with chronic heart failure	Gal-3 is useful as a marker for stratification for therapy (rosuvastatin and cardiac resynchronization) in patient with heart failure	
	HIV infection	Gal-3 is associated with early stages of HIV infection and is involved in the host response to HIV infection.			
I	Inflammation	Gal-3 is involved in many processes during both acute and chronic inflammatory response			
	Interstitial lung disease	Gal-3 is increased in the serum of patients affected by IPF, and is associated with decreased lung volumes and altered gas exchange			A novel target therapy for IPF was proposed using a Gal-3 inhibitor (TD139) formulated for inhalation and a clinical trial process was started
J	Juvenile Idiopathic Arthritis	Gal-3 serum levels are increased in children affected by JIA and correlate with disease activity			
K	Kidney	Gal-3 serum levels are upregulated in diabetic nephropathy and acute renal failure			Experimental evidence indicates that pharmacologic inhibition of Gal-3 with MCP is protective toward nephropathy. Another Gal-3 inhibitor, the GCS-100, significantly improved the eGFR in patients with CKD stage 3b.
L	Liver fibrosis	Gal-3 plays a critical role in hepatic stellate cells activation to a myofibroblast phenotype, a critical event in extracellular matrix deposition and in the development of cirrhosis			A Gal-3 antagonist, named GR-MD-02, is current on a phase 2 Clinical Trial to prevent progression toward cirrhosis in patients with NASH
M	Mortality		Gal-3 is associated with risk of coronary heart disease, CVD mortality, and all-cause mortality		
N	Non-alcoholic steatohepatitis (NASH)	Experimental evidence indicates that mice lacking the Gal-3 gene are resistant to liver fibrosis			The specific Gal-3 inhibitor, named GR-MB-02, is on Phase 2 Clinical Trial for the treatment of advanced fibrosis due to NASH
O	Obesity	Gal-3 is expressed in all types of macrophages that permeate white adipose tissue and it is an important regulator of their polarization and activity. Gal3 can bind directly to the insulin receptor (IR) and inhibit downstream IR signaling.			Experimental evidence in mice indicates that inhibition of Gal-3 improves insulin sensitivity
P	Pneumonia	Gal-3 is involved in the recruitment of neutrophils during lung infections with *Streptococcus pneumoniae*			Experimental evidence indicates that exogenous administration of Gal-3 to mice has a direct antimicrobial, bacteriostatic effect on *Streptococcus pneumonia*
	Pulmonary hypertension	Conflicting results:Gal-3 plasma levels are decreased in patients with PAH;Gal-3 plasma levels are increased in PAH independently to etiology and are are associated with severity of PAH			
	Plaque Psoriasis	Gal-3 is expressed in a high proportion of Langerhans cells from skin punch biopsies, obtained from lesional plaque-type psoriatic skin			An open-label phase 2a clinical trial is ongoing using the Gal-3 antagonist GR-MD-02 for the treatment of psoriasis
Q	Q Fever	Glactin-3 is a sensor of damage caused by the *Coxiella burnetii*			
R	Rheumatoid Arthritis	Gal-3 plays a key role as pro-inflammatory mediator in RA	Gal-3 serum level has been proposed as a predictive marker of future RA		Experimental evidence indicates that therapeutic intra-articularly administration of lentiviral vectors encoding Gal-3 small hairpin RNA (shRNA), in rats with collagen-induced arthritis, significantly ameliorates disease activity
S	Sepsis		Serum Gal-3 levels may represent a useful marker to predict mortality in patients affected by sepsis		Experimental evidence indicates that Gal-3 may represent a potential target for treatment of sepsis, at least for that induced by *Francisella novicida*
	Systemic Sclerosis	Serum Gal-3 levels correlate with the developmental process of skin sclerosis and of digital ulcers in diffuse cutaneous SSc	Gal-3 appears to be a reliable biomarker to predict all-cause mortality in patients affected by SSc		
T	Target therapy	Gal-3 is involved in numerous degenerative processes within the body, including cancer proliferation/metastasis, heart failure, atherosclerosis, diabetes, chronic inflammation, fibrosis and related organ failure.			Numerous compounds that are able to inhibit Gal-3 function have been proposed and are currently in Clinical Trials for many different diseases/conditions
U	Urinary tract infections	Gal-3 plays a role in urinary tract viral and bacterial infections in humans			
V	Venous Thrombosis	Serum levels of Gal-3 and of Gal-3 binding protein are elevated in the course of venous thrombosis			
W	Wound Healing	Gal-3 plays a key player in the re-epithelialization of wounds in many different tissues, including the cornea, the skin and the GI tract			
X	X syndrome of the heart	Serum Gal-3 levels are significantly higher in the CSX patients in comparison with the healthy controls			
Y	Yeast infection-Candidiasis	Gal-3 is present in the fungal granulomata, it binds to *Candida albicans* in a carbohydrate-specific manner and it displays a direct fungicidal activity			Experimental evidence indicates that the inhibition of Gal-3, obtained by knocking down the gene, by silencing its expression or by administration of the specific inhibitor TD139 is able to inhibit neutrophil reactive oxygen species (ROS) production and enhance the ability of neutrophils to kill *Candida albicans*
Z	Zoster-related pain (allodynia)	Gal-3 is involved in the process of Wallerian degeneration distal to the injury and subsequent axonal regrowth and functional re-innervation			Experimental evidence indicates that in mice the intrathecal administration of the Gal-3 inhibitor modified citrus pectin (MCP) is able to significantly attenuate neurophathic pain
